# USP1 promotes cholangiocarcinoma progression by deubiquitinating PARP1 to prevent its proteasomal degradation

**DOI:** 10.1038/s41419-023-06172-6

**Published:** 2023-10-11

**Authors:** Deng Yong Zhang, Yan Zhu, Qiong Wu, Shuoshuo Ma, Yang Ma, Zheng chao Shen, Zhonglin Wang, Wanliang Sun, Yong Chun Zhou, Dongdong Wang, Shuo Zhou, Zhong Liu, Lawrence N. Kwong, Zheng Lu

**Affiliations:** 1 Department of General Surgery, The First Affiliated Hospital of Bengbu Medical College, Bengbu, 233000 Anhui China; 2 Department of Translational Molecular Pathology, The University of Texas MD Anderson Cancer Center, Houston, TX 77030 USA; 3 Anhui Medical university, Hefei, 230000 Anhui China; 4 Cancer Biology Program, The University of Texas MD Anderson Cancer Center UTHealth Graduate School of Biomedical Sciences, Houston, TX 77030 USA; 5 Department of pathology, The First Affiliated Hospital of Bengbu Medical College, Bengbu, 233000 Anhui China; 6 Department of pharmacy, Bengbu Medical College, No.2600 Donghai Road, Bengbu, 233000 Anhui China; 7 Department of General Surgery, The First Affiliated Hospital of Wannan Medical College, Wuhu, 241001 Anhui China; 8 Social Science Research Institute, Duke University, Durham, NC 27708 USA; 9 Department of Radiotherapy, The First Affiliated Hospital of Bengbu Medical College, Anhui, China; 10 Department of Genomic Medicine, The University of Texas MD Anderson Cancer Center, Houston, TX 77030 USA

**Keywords:** Bile duct cancer, Acetylation

## Abstract

Despite its involvement in various cancers, the function of the deubiquitinase USP1 (ubiquitin-specific protease 1) is unexplored in cholangiocarcinoma (CCA). In this study, we provide evidence that USP1 promotes CCA progression through the stabilization of Poly (ADP-ribose) polymerase 1 (PARP1), consistent with the observation that both USP1 and PARP1 are upregulated in human CCA. Proteomics and ubiquitylome analysis of USP1-overexpressing CCA cells nominated PARP1 as a top USP1 substrate. Indeed, their direct interaction was validated by a series of immunofluorescence, co-immunoprecipitation (CO-IP), and GST pull-down assays, and their interaction regions were identified using deletion mutants. Mechanistically, USP1 removes the ubiquitin chain at K197 of PARP1 to prevent its proteasomal degradation, with the consequent PARP1 stabilization being necessary and sufficient to promote the growth and metastasis of CCA in vitro and in vivo. Additionally, we identified the acetyltransferase GCN5 as acetylating USP1 at K130, enhancing the affinity between USP1 and PARP1 and further increasing PARP1 protein stabilization. Finally, both USP1 and PARP1 are significantly associated with poor survival in CCA patients. These findings describe PARP1 as a novel deubiquitination target of USP1 and a potential therapeutic target for CCA.

## Introduction

CCA originates from the epithelial cells of the biliary tract, accounting for 3% of digestive system cancers. CCA often presents with nonspecific symptoms and is frequently diagnosed at an advanced stage with metastasis to distant organs, precluding curative surgical intervention. Currently, there are no effective therapies for patients with advanced-stage CCA, resulting in a dismal 5-year survival rate of only 7–20% [1, 2].

The significance of protein degradation in cancer is increasingly evident, yet its relevance to CCA remains understudied. Typically, protein degradation involves two classical pathways: the ubiquitin-proteasome pathway and the autophagy-lysosome pathway[3, 4]. Ubiquitination not only targets proteins for proteasomal degradation and reduces their stability, but also modulates protein function by altering their interactions with other proteins [5, 6]. As a pivotal member of the deubiquitinating enzyme family, USP1 exerts regulatory effects on various oncological biological processes, including tumor proliferation, drug resistance, and metastasis [7–10]. USP1 acts as a deubiquitinating enzyme in various cancers including lung cancer and hepatocellular carcinoma, regulating downstream target proteins and impacting tumor progression[11–13]. While USP1’s function remains conservative across different types of cancer, its downstream targets may vary. Therefore, understanding the specific targets and effects of USP1 in each cancer type could lead to innovative therapeutic opportunities.

In addition to ubiquitination, acetylation is another type of protein modification that occurs on lysine residues and plays a crucial role in regulating various cellular functions, including but not limited to protein-protein interactions, transcriptional regulation, subcellular localization, and enzymatic activities. Acetylation also participates in the regulation of protein stability by competing with ubiquitination for the same receptor lysine residue [14] or by facilitating the formation or dissociation of protein complexes from E3s and deubiquitinases (DUB), thereby resulting in the degradation or stabilization of proteins [15].

We demonstrate that both ubiquitination and acetylation are crucial for the activity of USP1 in CCA. Firstly, we identify the deubiquitination of PARP1 by USP1 as one of its major targets in CCA. Secondly, we discover that GCN5 acetylates USP1 to enhance the affinity between USP1 and PARP1, thereby further stabilizing PARP1. Moreover, USP1 is upregulated in cholangiocarcinoma (CCA) and its positive regulation of PARP1 leads to increased proliferation, invasion, and metastasis in CCA models. Our findings suggest that PARP1 represents a novel deubiquitinating target of USP1 and thus a potential therapeutic target for the treatment of CCA.

## Results

### PARP1 is a major target of USP1 deubiquitinase activity in CCA

**Fig. 1:**
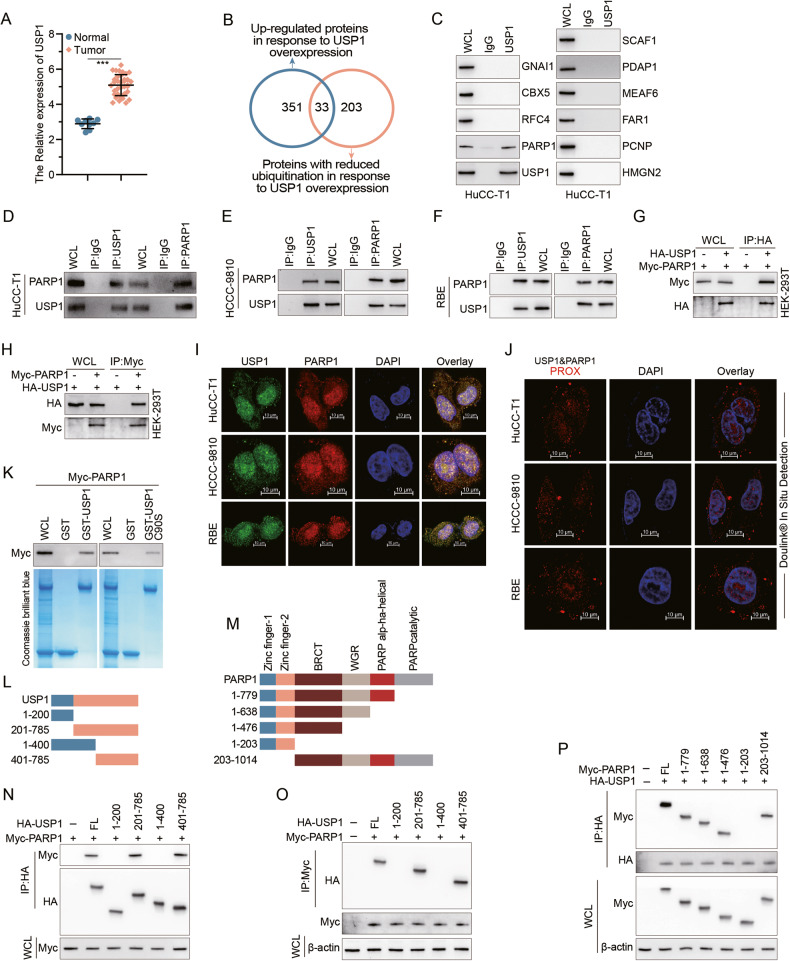
USP1 interacts with PARP1. **A** The expression of USP1 in CCA in TCGA databasae. **B** Proteomics and mass spectrometry analysis of ubiquitination were conducted in RBE cells with stable USP1 overexpression, resulting in the identification of proteins exhibiting reduced ubiquitination levels and increased expression. IgG was used as the isotype control for CO-IP assay. **C** CO-IP assay was conducted to identify the interaction between USP1 and the 10 candidate proteins identified in B in HuCC-T1. WCL: whole cell lysates. **D**, **F** Cell lysates from HuCC-T1 (**D**), HCCC-9810 (**E**), and RBE (**F**) were analyzed by IP using antibodies against USP1 and PARP1, then subjected to IB analysis, respectively. IgG was used as the isotype control. **G**, **H** HA-USP1 and Myc-PARP1 were co-transfected into HEK-293T cells. The cell lysates were subjected to IP with anti-HA or anti-Myc antibodies followed by IB analysis with antibodies against Myc and HA. **I** HuCC-T1, HCCC-9810, and RBE cells were fixed and stained with USP1 antibody (Green) and PARP1 antibody (Red). Nuclei were stained with DAPI (blue). Scale bar: 10 μm. **J** After fixation, in situ PLA for USP1/PARP1 was performed with anti-USP1 and anti-PARP1 antibodies. The PLA-detected proximity (PROX) complexes are represented by the fluorescent rolling circle products (red dots). Scale bars, 10 mm. **K** Purified Myc-PARP1 was incubated with GST, GST-USP1, or GST-USP1 C90S coupled to glutathione-Sepharose beads. Proteins retained on Sepharose were then subjected to IB analysis with indicated antibodies. Recombinant GST-USP1 and GST-USP1 C90S was purified from bacteria and analyzed by SDS-PAGE and Coomassie blue staining. **L**, **M** Schematic representation of HA-tagged full-length (FL) USP1, Myc-tagged FL PARP1, and their various deletion mutants. **N**, **O** HEK-293T cells were co-transfected with Myc-PARP1 and HA-tagged FL USP1 or its deletion mutants, and cell lysates were analyzed by IP with HA (**N**) or Myc (**O**) beads followed by IB analysis with antibodies against Myc and HA. **P** HEK-293T cells were co-transfected with HA-USP1 and Myc-tagged FL PARP1 or its deletion mutants, and cell lysates were analyzed by IP with HA beads followed by IB analysis with antibodies against HA and Myc.

To elucidate the potential involvement of USP1 in the initiation and progression of CCA, we first identified a significant upregulation of USP1 in CCA tissues compared to adjacent in the TCGA database (Fig. 1A). Next, we established a stable RBE cell line overexpressing USP1 and conducted both mass spectrometer-based total and ubiquitination-specific proteomics analyses (Fig. 1B). Given the deubiquitinating function of USP1, our focus was on identifying proteins that were upregulated upon USP1 overexpression and which also exhibited reduced levels of deubiquitination. We identified 384 upregulated proteins and 236 proteins with reduced ubiquitination (Supplementary Tables 4 and 5), of which 33 were present in both groups. Combine with the results of mass spectrome, we selected the Top 10 candidates and tested them individually using IP assay. Of these, only PARP1 (Fig. 1C, Sup. Fig. 1A) validated as interacting with USP1. We therefore focused on PARP1, a well-known cancer-associated protein that undergoes PTM (post-translational modification) in many cancer types [16–18], for subsequent mechanistic studies.

To expand the validation of the observed USP1-PARP1 interaction, we performed confirmatory CO-IP assays in the CCA cell lines HuCC-T1, HCCC-9810, RBE, and HEK-293T (Fig. 1D–H). Immunofluorescence staining confirmed their co-localization mainly within the cell nucleus, with a small fraction distributed in the cytoplasm of HuCC-T1, HCCC-9810, and RBE cells (Fig. 1I). In order to gain higher specificity and sensitivity, we also performed a proximity ligation assay (PLA) using primary antibodies targeting USP1 and endogenous PARP1, followed by secondary antibodies labeled with specific detection oligonucleotides. We observed a predominant PLA signal localized in both the cell nucleus and cytoplasm (Fig. 1J, Supplementary Fig. 1B), further supporting a direct USP1-PARP1 interaction. In vitro GST pull-down experiments using the recombinant proteins GST-USP1 or a catalytically inactive mutant GST-USP1 C90S revealed that both purified proteins can bind to Myc-PARP1, while GST alone cannot under cell-free conditions (Fig. 1K), indicating that their direct interaction is independent of USP1’s enzymatic activity.

To investigate the specific region responsible for the interaction between USP1 and PARP1, we generated truncated mutant fragments of both proteins (Fig. 1L, M) to pinpoint the binding sites. Our transfection experiments in HEK-293T cells revealed that deletion of amino acids 201-785 and 401-785 from USP1 abolished its ability to bind to PARP1, while deletion of amino acids 1–200 or 1–400 had no effect (Fig. 1N, O), indicating that the critical region for PARP1 binding is between amino acids 401–785 of USP1. For PARP1, deletion of amino acids 203-1014 abolished binding to USP1, whereas loss of amino acids 1–203 or a series of C-terminal truncations retained binding, including deletion of amino acids 476–1014; together, these narrowed down the critical USP1-binding region to amino acids 203-476 of PARP1 (Fig. 1P, Sup. Fig. 1C), which contains the known protein-protein interaction-mediating BRCT domain. These experiments delineate the critical reciprocal binding regions of each protein.

### USP1 prevents PARP1 protein degradation

**Fig. 2:**
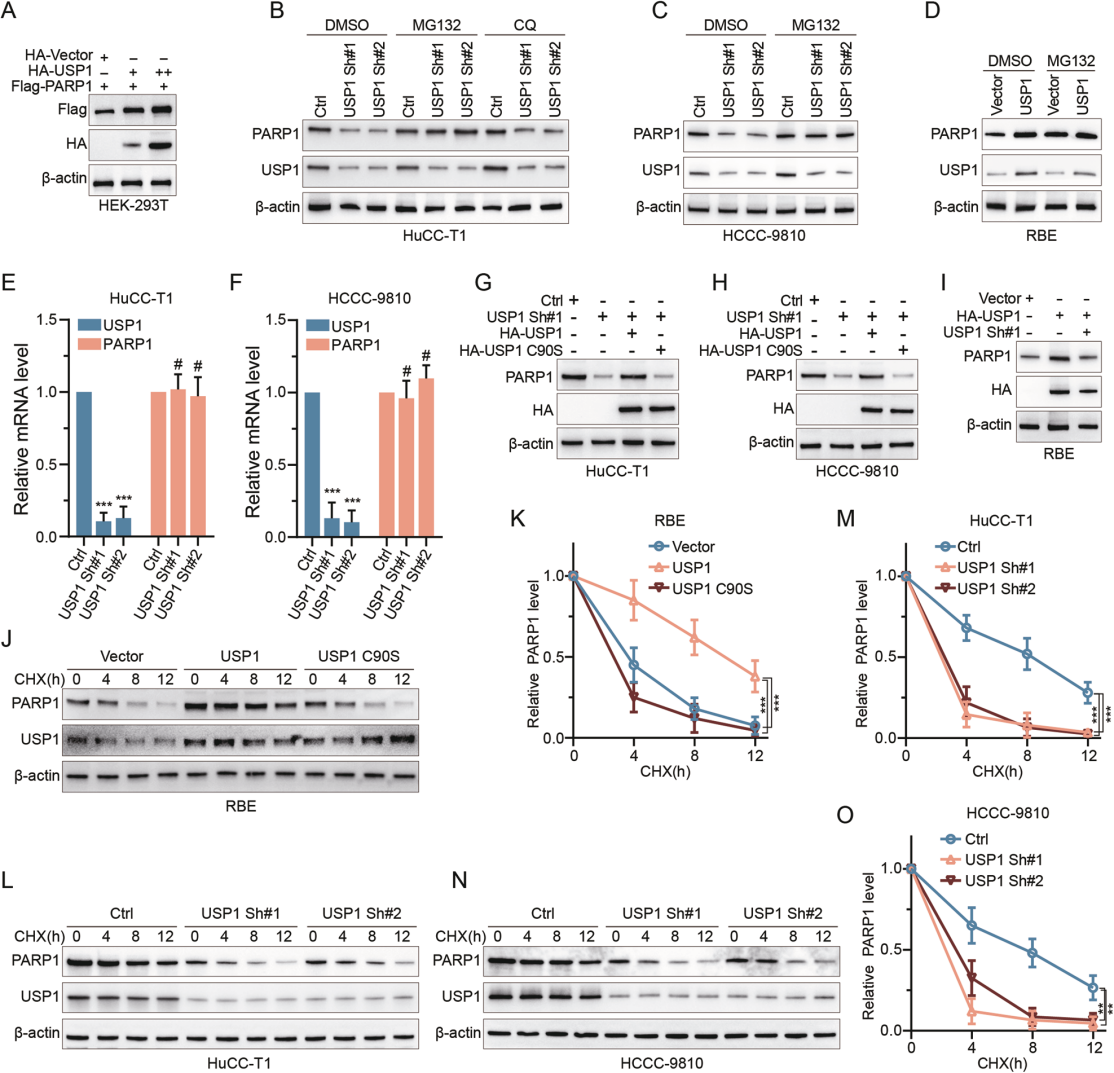
USP1 enhances the stability of PARP1. **A** Increasing amounts of USP1 were transfected into HEK-293T cells and PARP1 expression was detected by IB analysis. **B**, **C** HuCC-T1 and HCCC-9810 cells transfected with two independent USP1 shRNA were treated with or without the proteasome inhibitor MG132 (20 μM, 8 hours) and autophagy inhibitor CQ (25 μM, 2 hours), and then USP1 and PARP1 were analyzed. **D** Detection of PARP1 protein level by IB analysis after treatment with DMSO or proteasome inhibitor MG132 (20 μM) for 8 hours in RBE transfected with vector or USP1. **E**, **F** The qRT-PCR was used to detect the mRNA level of USP1 and PARP1 in HuCC-T1 (**E**) and HCCC-9810 (**F**) cell lines transfected with the indicated shRNAs. **G**, **H** IB analysis was conducted to assess PARP1 protein levels in HuCC-T1 and HCCC-9810 cells with stable USP1 knockdown, combined with overexpression of either HA-USP1 or HA-USP1 C90S, respectively. **I** IB analysis of PARP1 was conducted in RBE cells overexpressing USP1 or with combined knockdown of USP1. **J**, **K** RBE cells were transfected with USP1 or USP1 C90S, treated with 50 μg/ml CHX, collected at the indicated times, and then subjected to IB analysis with antibodies against PARP1 and USP1. Quantification of PARP1 levels relative to β-actin is shown (**K**). **L**–**O** HuCC-T1 (**L**, **M**) and HCCC-9810 (**N**, **O**) cells stably expressing control shRNA or two independent USP1 shRNA were treated with 50 μg/ml CHX, harvested at the indicated times, and then subjected to IB analysis with antibodies against PARP1 and USP1. Quantification of PARP1 levels relative to β-actin is shown. The data were obtained from three independent biological replicates and are presented as mean ± SD. One-way ANOVA with Dunnett’s post test (**E**, **F**, **K**, **M**, **O**). ***p < 0.001, **p < 0.005, ^#^*p* > 0.05.

To validate the hypothesis that USP1 directly stabilizes PARP1 by preventing its degradation, we first overexpressed USP1 in HEK-293T cells at two dose levels and observed a corresponding gradient increase in PARP1 protein levels (Fig. 2A). In CCA cell lines, knockdown of USP1 resulted in decreased PARP1 protein levels while USP1 overexpression had the opposite effect. Next, we found that the proteasome inhibitor MG132 reversed the decrease in PARP1 protein levels caused by USP1 knockdown, but not the increase in PARP1 protein caused by USP1 overexpression (Fig. 2B–D, Sup. Fig. 2A, B). By contrast, the autophagosome pathway inhibitor CQ failed to reverse the effects of USP1 knockdown on PARP1 (Fig. 2B). Through qRT-PCR analysis, we observed that USP1 knockdown or overexpression did not exert any significant effect on the mRNA levels of PARP1 (Fig. 2E, F, Sup. Fig. 2C).

Next, we found that USP1 knockdown-mediated PARP1 protein reduction was almost completely restored upon overexpression of USP1, but not by the catalytically inactive mutant USP1 C90S (Fig. 2G–I). Finally, we administered the protein synthesis inhibitor CHX to RBE cells overexpressing either USP1 or USP1 C90S. Only overexpression of wild-type USP1 prevented PARP1 protein degradation (Fig. 2J–K). Additionally, in HuCC-T1 and HCCC-9810 cells, CHX treatment similarly reduced PARP1 protein levels, which was further exacerbated by the knockdown of USP1 (Fig. 2L–O). Taken together, these results indicate that USP1 regulates PARP1 post-translationally through the prevention of proteosomal degradation, and that this requires USP1 catalytic activity.

### USP1 prevents PARP1 degradation through deubiquitination

**Fig. 3:**
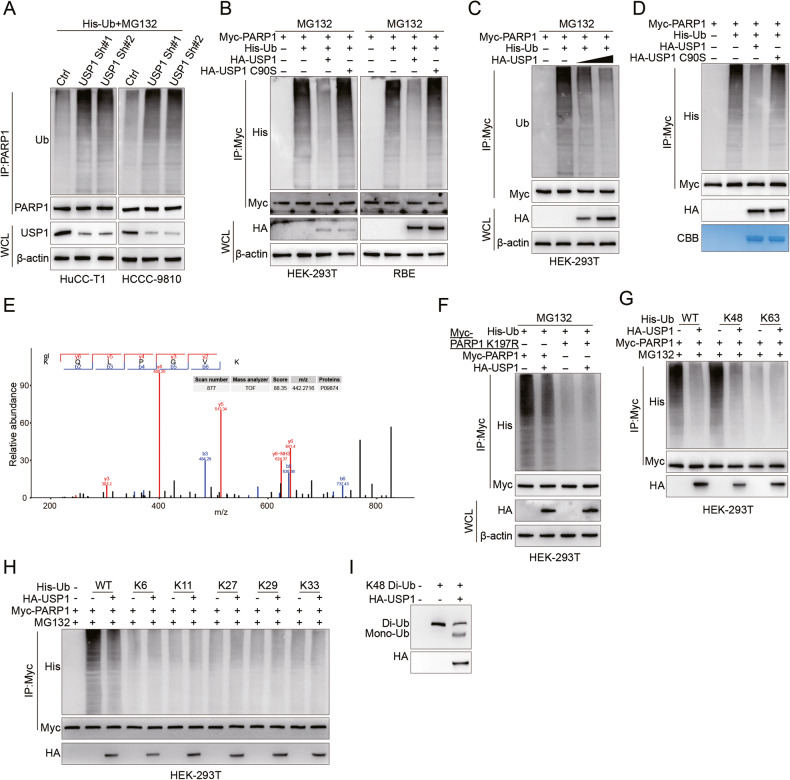
USP1 removes the K48-linked ubiquitin chain from K197 of PARP1. **A** HuCC-T1 and HCCC-9810 were co-transfected with the indicated ShRNA and His-Ub, and cell lysates were subjected to IP with PARP1 antibody, followed by IB analysis with antibodies against His and PARP1. Cells were treated with 20 μM MG132 for 8 hours before harvesting. **B** HEK-293T or RBE cells were co-transfected with Myc-PARP1, His-Ub, and HA-USP1 or USP1 C90S, and cell lysates were subjected to IP with Myc beads followed by IB analysis with antibodies against His and Myc. Cells were treated with 20 μM MG132 for 8 hours before harvesting. **C** HEK-293T cells was co-transfected with Myc-PARP1, His-Ub, and Increasing amounts of HA-USP1, and cell lysates were subjected to IP with Myc beads followed by IB analysis with antibodies against His and Myc. Cells were treated with 20 μM MG132 for 8 hours before harvesting. **D** Ubiquitylated Myc-PARP1 was incubated with purified HA-USP1 or HA-USP1 C90S from HEK-293T cells. Detection of Myc-PARP1 and His-Ub was performed by IB analysis using anti-Myc and anti-His antibodies, respectively. The purity of the purified HA-USP1 and HA-USP1 C90S proteins was assessed by SDS-PAGE followed by Coomassie blue staining. **E** Ubiquitination mass spectrometry of the PARP1 peptide in RBE cell with overexpressed USP1. **F** HEK-293T cells were co-transfected with HA-USP1, His-Ub, and Myc-PARP1 or Myc-PARP1-K197R, and cell lysates were subjected to IP with Myc beads followed by IB analysis with antibodies against His and Myc. Cells were treated with 20 μM MG132 for 8 hours before harvesting. **G** HEK-293T cells were co-transfected with Myc-PARP1, HA-USP1, and the indicated His-Ub WT, or His-K48-Ub (Lys48-only), or His-K63-Ub (Lys63-only) plasmids, and then the PARP1 ubiquitylation linkage was analyzed by IB analysis using anti-His antibody. **H** HEK-293T cells were co-transfected with Myc-PARP1, HA-USP1, and the indicated His-Ub WT, or His-K6-Ub (Lys6-only), or His-K11-Ub (Lys11-only), or His-K27-Ub (Lys27-only), or His-K29-Ub (Lys29-only), or His-K33-Ub (Lys33-only) plasmids, and then the PARP1 ubiquitylation linkage was analyzed by IB analysis using anti-His antibody. **I** Purified HA-USP1 was incubated with K48-linked di-ubiquitin in the presence of the compound at 37 °C for 1.5 hours. Samples were then analyzed by IB analysis using an anti-Ub antibody.

Consistent with USP1 directly deubiquitinating PARP1 in CCA, we observed an increase in endogenous ubiquitination levels of PARP1 upon knockdown of USP1 in HuCC-T1 and HCCC-9810 cells (Fig. 3A). Conversely, transfection of HA-USP1, but not HA-USP1 C90S, resulted in a decrease in exogenous PARP1 ubiquitination levels in HEK-293T and RBE cells (Fig. 3B). Moreover, as the dosage of USP1 increased, the ubiquitination level of PARP1 similarly decreased further (Fig. 3C). To determine whether PARP1 is a direct deubiquitination substrate of USP1, we co-incubated poly-ubiquitinated PARP1 with purified HA-USP1 or HA-USP1 C90S under cell-free conditions. HA-USP1, but not HA-USP1 C90S, specifically removed the poly-ubiquitination chain of PARP1 (Fig. 3D). Together, these results confirm that USP1 directly deubiquitinates PARP1.

To determine the lysine site of PARP1 targeted by USP1, we conducted a thorough analysis of the ubiquitination-specific mass spectrometry data and observed that Lys-197 may be an important site for USP1 deubiquitination of PARP1 (Fig. 3E). We also found that USP1 did not deubiquitinate PARP1-K197R (Fig. 3F). It suggests that K197 site is the primary USP1 deubiquitination target on PARP1 controlling its degradation.

Poly-ubiquitination chains are generated primarily through two distinct types of bonds: Lys48 or Lys63 chains. The Lys48-linked ubiquitin chain serves as the principal target signal for proteasomal degradation, whereas the Lys63-linked ubiquitin chain participates in a diverse range of proteasome-independent cellular event [19, 20]. By using specific ubiquitin mutants, we determined that USP1 effectively cleaves lys48-linked PARP1 poly-ubiquitination, but not lys63-linked PARP1 poly ubiquitination (Fig. 3G). Additionally, USP1 does not significantly affect the ubiquitination of PARP1 linked by lys6, lys11, lys27, lys29 and lys33 (Fig. 3H). In an in vivo di-ubiquitination formation experiment using ubiquitin chains as substrates, we observed USP1’s ability to cleave K48-linked di-ubiquitin (Fig. 3I). In summary, these findings demonstrate that USP1 functions as a deubiquitinating enzyme targeting K197 on PARP1.

### USP1 enhances the proliferation, invasion, and metastasis of CCA by stabilizing PARP1

**Fig. 4:**
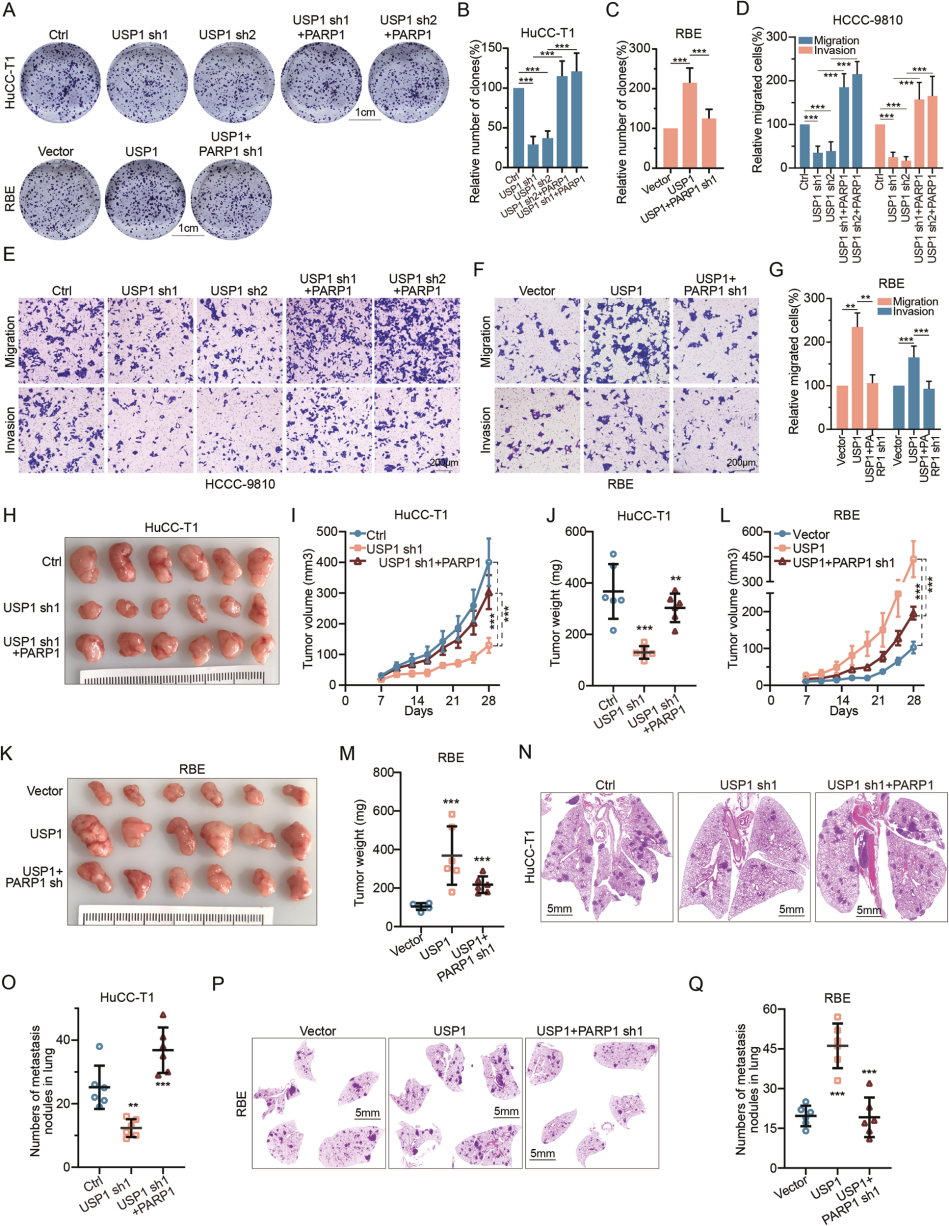
USP1 promotes CCA proliferation, invasion, and metastasis through PARP1. **A**–**C** Colony assay was assessed in HuCC-T1 transfected with shCtrl, USP1-sh1, or USP1-sh2, reconstituted with PARP1. Additionally, colony assay was conducted on RBE cells transfected with either vector or USP1 and reconstituted with PARP1 sh1. Representative images are presented in **A**, while the relative number of clones is quantified in **B**, **C**. **D**, **E** The Transwell assay was performed on HCCC-9810 cells transfected with shCtrl, USP1-sh1 or USP1-sh2, and reconstituted with PARP1. Representative images are presented in **E**, while the relative number of migrated cells was quantified **D**. **F**, **G** The Transwell assay was performed on RBE cells transfected with either vector or USP1 and reconstituted with PARP1 sh1. Representative images are presented in **F**, while the relative number of migrated cells was quantified in **G**. **H**–**J** The Xenograft tumor formation assay was performed on HuCC-T1 cells transfected with shCtrl, USP1-sh1, and reconstituted with PARP1. The indicated cells were subcutaneously injected (2.5 × 10^6^ cells per mouse) into nude mice (*n* = 6). Gross images of the tumors were as shown in **H**. The growth curve of the tumor is shown in **I**. The weight of the tumors was shown in **J**. **K**–**M** The Xenograft tumor formation assay was performed on RBE cells transfected with either vector or USP1 and reconstituted with PARP1 sh1. The indicated cells were subcutaneously injected (2.5 × 10^6^ cells per mouse) into nude mice (*n* = 6). Gross images of the tumors were as shown in K. The growth curve of the tumor was shown in L. The weight of the tumors was shown in **M**. **N**–**O** The lung metastasis model assay was performed on HuCC-T1 cells transfected with shCtrl, USP1-sh1, and reconstituted with PARP1. Representative images are shown in **N**, and the relative number of lung metastases is shown in **O**. **P**, **Q** The lung metastasis model assay was performed on RBE cells transfected with either vector or USP1 and reconstituted with PARP1 sh1. Representative images are shown in **P**, and the relative number of lung metastases is shown in **Q**. Data are from three independent biological repeats and presented as mean ± SD. ***P < 0.001, **p < 0.005, one-way ANOVA with Dunnett’s post test.

To assess the functional implications of USP1-mediated regulation of PARP1 on CCA phenotypes, we initially silenced USP1 in HuCC-T1 and HCCC-9810 cells, resulting in suppressed cellular proliferation in vitro. Conversely, upregulation of USP1 increased proliferation in RBE cells. Critically, both effects can be reversed through the overexpression or knockdown of PARP1, respectively (Fig. 4A–C, Sup. Fig. 3A, B). Furthermore, suppression of USP1 impedes the invasion of HCCC-9810 and HuCC-T1, whereas upregulation of USP1 promotes invasion in RBE cell line. Again, these can be reversed by modulating PARP1 expression in the opposite direction (Fig. 4D–G, Sup. Fig. 3C). We also detected the apoptosis of CCA cells by flow cytometry, and found that the percentage of cell apoptosis increased after USP1 was knocked out in HuCC-T1, and decreased after overexpression of USP1 in RBE, both effects can be reversed through the overexpression or knockdown of PARP1, respectively (Sup. Fig. 3D).

Next, we found that USP1 knockdown impeded the in vivo growth of HuCC-T1 xenografts, while USP1 overexpression enhanced the growth of RBE xenografts. Similar to in vitro, both outcomes could also be reversed by either overexpressing or knocking down PARP1, respectively (Fig. 4H–M). Moreover, using an experimental tail vein lung metastasis protocol, USP1 knockdown led to a reduction in the number of metastases, while USP1 overexpression led to an increase, again dependent on PARP1 overexpression or knockdown, respectively (Fig. 4N, Q). Collectively, these findings indicate that USP1 can promote CCA proliferation and metastasis primarily through PARP1 both in vitro and in vivo.

### GCN5 induces acetylation of USP1 to strengthen PARP1 stability

**Fig. 5:**
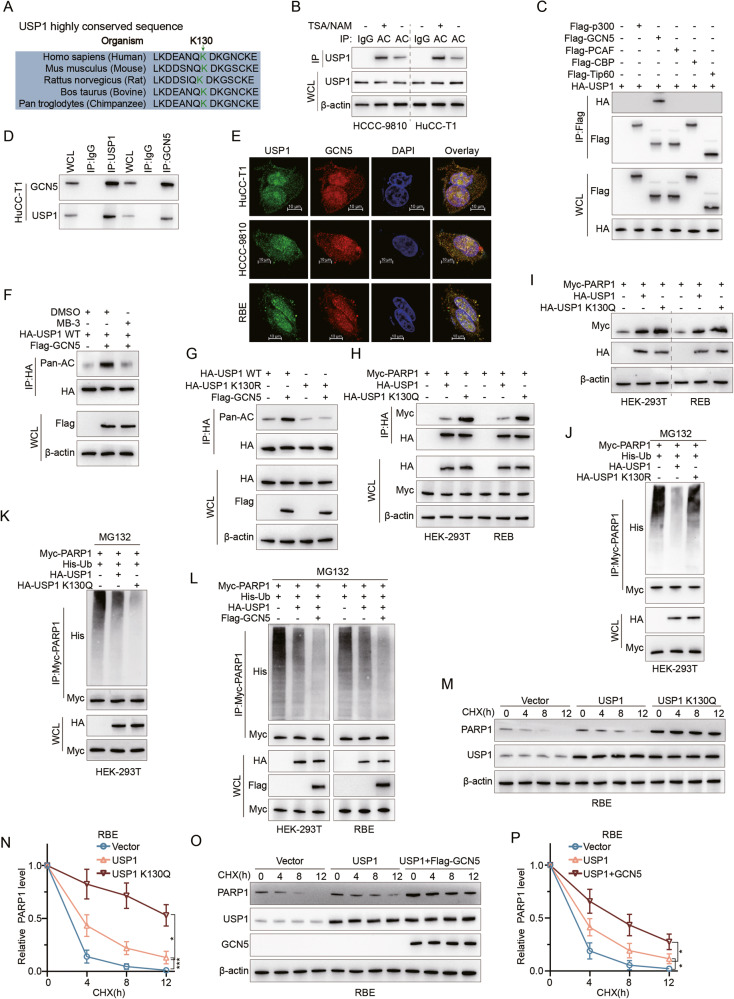
GCN5 induces acetylation of USP1 to strengthen PARP1 stability. **A** Sequence alignment of the conserved K130 containing region in USP1 orthologs of different species. **B** Nicotinamide (NAM) (5 mM, 4 hours) and trichostatin A (TSA) (0.5 μM, 16 hours) were used to improve protein acetylation levels, and acetylated USP1 in HuCC-T1 and HCCC-9810 was IP with pan-acetylation (Pan-AC) antibody followed by IB analysis with antibodies against USP1. **C** In HEK-293T cells, HA-USP1 was co-expressed with Flag-tagged P300, GCN5, PCAF, CBP, or Tip60 acetyltransferases respectively. The cell lysates were subjected to IP with anti-Flag followed by IB analysis with antibodies against HA and Flag. **D** Cell lysates from HuCC-T1 cells were analyzed by IP using antibodies against USP1 and GCN5, then subjected to IB analysis. **E** HuCC-T1, HCCC-9810, and RBE cells were fixed and stained with USP1 antibody (Green) and GCN5 antibody (Red). Nuclei were stained with DAPI (blue). Scale bar: 10 μm. **F** HA-USP1 WT and Flag-GCN5 were co-expressed in HEK-293T cells with or without MB-3. The cell lysates were subjected to IP with anti-HA followed by IB analysis with antibodies against HA, Pan-AC, and Flag. **G** Flag-GCN5 and HA-USP1 WT or HA-USP1 K130R mutants were co-overexpressed in HEK-293T cells. The cell lysates were subjected to IP with anti-HA followed by IB analysis with antibodies against HA, Pan-AC, and Flag. **H** Myc-PARP1 and HA-USP1 or HA-USP1 K130Q mutants were co-overexpressed in HEK-293T and RBE cells. The cell lysates were subjected to IP with anti-HA followed by IB analysis with antibodies against HA and Myc. **I** IB analysis of Myc-PARP1 was conducted in RBE and HEK-293T cells overexpressing HA-USP1 or HA-USP1 K130Q. **J** IB analysis of total lysates and anti-Myc immunoprecipitates of HEK-293T cells transfected with His-Ub, HA-USP1/K130R, or Myc-PARP1 constructs and treated with MG132 (20 μM for 8 hours) as indicated. **K** IB analysis of total lysates and anti-Myc immunoprecipitates of HEK-293T cells transfected with His-Ub, HA-USP1/K130Q, or Myc-PARP1 constructs and treated with MG132 (20 μM for 8 hours) as indicated. **L** IB analysis of total lysates and anti-Myc IP of HEK-293T and RBE cells transfected with His-Ub, HA-USP1/K130R, Flag-GCN5 or Myc-PARP1 constructs and treated with MG132 (20 μM for 8 hours) as indicated. **M**, **N** RBE cells were transfected with USP1 or USP1 K130Q, treated with 50 μg/ml CHX, collected at the indicated times, and then subjected to IB analysis with antibodies against PARP1 and USP1. Quantification of PARP1 levels relative to β-actin is shown (**M**). **O**, **P** With or without Flag-GCN5, RBE cells were transfected with either USP1 WT or USP1 K130Q, treated with 50 μg/ml CHX, collected at designated time points, and subsequently subjected to IB analysis using antibodies against PARP1 and USP1. The quantification of PARP1 levels relative to β-actin is presented. The data were obtained from three independent biological replicates and are presented as mean ± SD. One-way ANOVA with Dunnett’s post test (**M**, **O**). ***P < 0.001, *p < 0.05.

We next asked whether USP1 itself is regulated by PTM. After examining the proteomic databases PhosphoSitePlus, we discovered that USP1 possesses multiple acetylation sites (Sup. Fig. 4A), including K130, which is highly conserved across different species (Fig. 5A). Upon treatment with deacetylase inhibitors (TSA/NAM), IP analyses revealed a significant increase in acetylation of USP1 in HuCC-T1 and HCCC-9810 (Fig. 5B). Generally speaking, five acetyltransferases including P300, GCN5, PCAF, CBP and Tip60 mediate about 90% of protein acetylation in eukaryotes [21, 22]. A systematic IP experiment using all 5 acetyltransferases identified GCN5 as the only interactor with USP1 in HEK-293T cells (Fig. 5C). This direct interaction was confirmed in CCA cells through IP and immunofluorescence co-localization assays (Fig. 5D, E and Sup. Fig. 4B). We also found that GCN5 was highly expressed in most CCA patients by western blot and IHC assay (Sup. Fig. 4C, D).

We next overexpressed GCN5 in HEK-293T cells and observed an increase in USP1 acetylation, whereas treatment with the GCN5 inhibitor MB-3 reduced USP1 acetylation (Fig. 5F). We further focused on the K130 site on USP1 by creating a non-acetylatable USP1-K130R mutant. Notably, upon GCN5 overexpression, the overall acetylation level of the USP1-K130R mutant did not increase in HEK-293T cells (Fig. 5G). Consistent with this, overexpression of the acetyl-mimetic K130Q USP1 in HEK-293T and RBE cell lines showed a stronger affinity between USP1 K130Q and PARP1 compared to wild-type USP1 as well as a stronger increase in PARP1 protein levels (Fig. 5H, I), suggesting that K130 acetylation positively regulates USP1 function. Then we find that USP1 K130R overexpression led to a higher increase in PARP1 ubiquitination compared to wild-type USP1 (Fig. 5J). And vice versa, USP1 K130Q overexpression led to low ubiquitination of PARP1 (Fig. 5K). Moreover, co-overexpression of GCN5 enhanced the effect of USP1 overexpression on PARP1 deubiquitination in both HEK-293T and RBE cells (Fig. 5L). Finally, using the CHX protein synthesis inhibition assay, we found that overexpression of USP1 K130Q in RBE significantly enhanced the stability of endogenous PARP1 protein levels compared to wild-type USP1 (Fig. 5M, N). Similarly, co-overexpression of GCN5 with USP1 also strengthens the stability of PARP1 protein levels (Fig. 5O, P). Together, our findings establish GCN5 as the primary USP1 acetylase, targeting K130 and thereby enhancing USP1 function and PARP1 stability.

### The expression of USP1 correlates with PARP1 in CCA patients

**Fig. 6:**
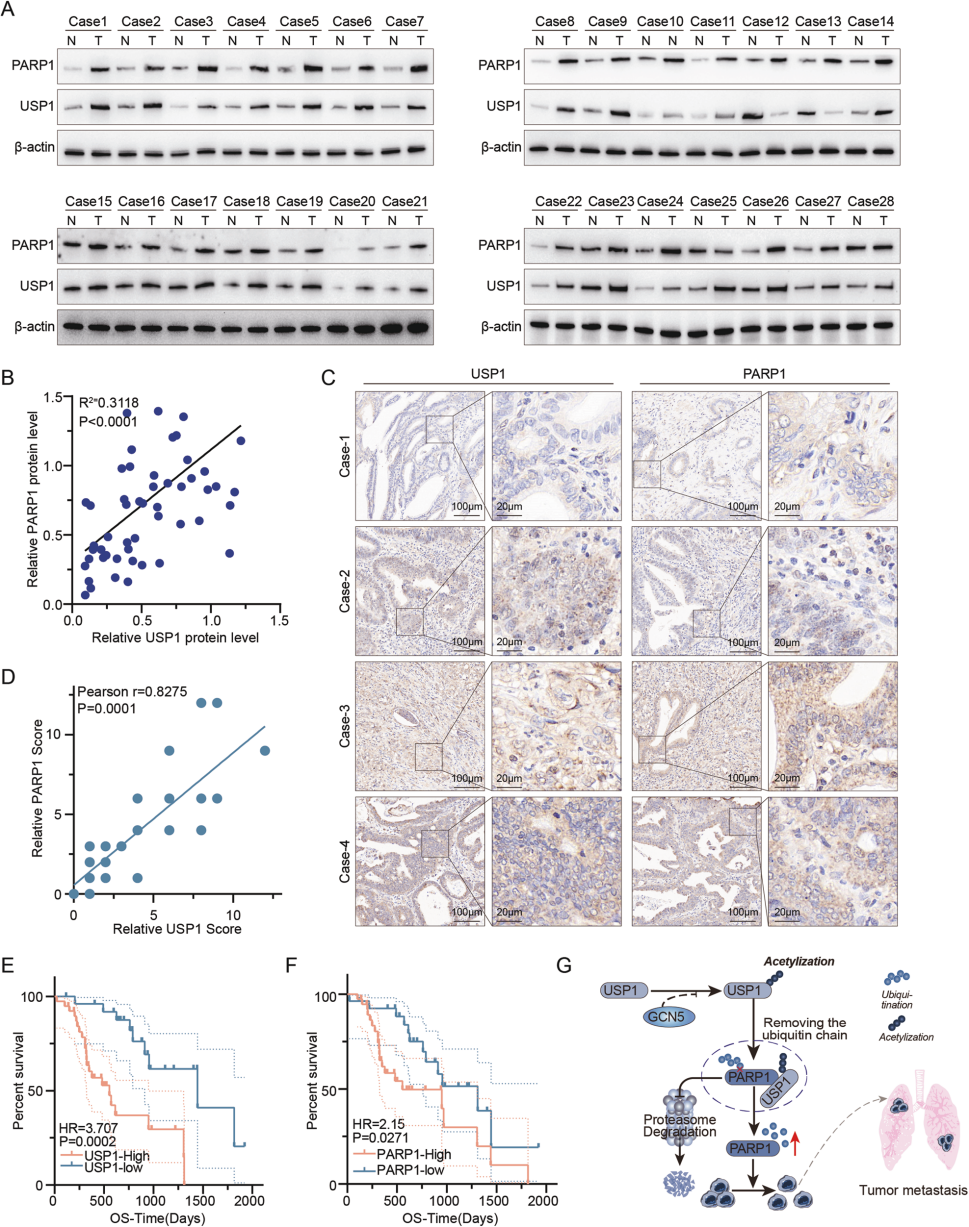
USP1 is enriched in CCA tissues and its expression correlates negatively with patients’ survivals. **A** IB analysis of cell lysates from 28 CCA samples using USP1 and PARP1 antibodies. **B** Correlation analysis of USP1 and PARP1 in CCA samples (*n* = 28). Chi-square test was used for statistical analysis. Pearson r represents the correlation coefficient. **C** Representative images of IHC staining of USP1 and PARP1 on tissue of CCA patients (*n* = 65). Scale bars are indicated. **D** Correlation analysis of IHC-scores of USP1 and PARP1 in CCA samples (*n* = 65). Chi-square test was used for statistical analysis. Pearson *r* represents the correlation coefficient. **E** Kaplan–Meier curves showing overall survival of CCA patients divided based on USP1 expression in tumors (*P* values by log-rank test). **F** Kaplan-Meier curves showing overall survival of CCA patients divided based on PARP1 expression in tumors (*P* values by log-rank test). **G** The graphic summary created by Adobe Illustrator, depicts the regulation of PARP1 by USP1. Specifically, GCN5 acetylates USP1 at K130 to enhance its affinity for PARP1. Additionally, USP1 stabilizes PARP1 protein by removing the ubiquitin chain at K197 and promotes CCA progression.

To assess the clinical significance of the USP1-PARP1 axis in CCA, we first examined the relationship between USP1 and PARP1 protein expression in human CCA samples. Through immunoblot analysis, a positive correlation was observed between USP1 and PARP1 protein levels in CCA samples (*n* = 28, *P* < 0.0001, Pearson *r* = 0.3118) (Fig. 6A, B). Subsequently, IHC staining for USP1 and PARP1 was performed on CCA samples (*n* = 65). Representative images stained for USP1 and PARP1 are presented in Fig. 6C, D, demonstrating a significant positive correlation between the two proteins (Pearson *r* = 0.0002, *P* = 0.1987). Kaplan–Meier survival analysis (*n* = 65) demonstrated a significant correlation between the upregulation of either USP1 or PARP1 expression with a shortened overall survival (Fig. 6E, F). These findings collectively suggest that USP1 regulates PARP1 in CCA patients and that both are significantly associated with an unfavorable prognosis. Figure 6G show the overall design of this study, which showed that GCN5 acetylated USP1 at K130, enhancing the affinity between USP1 and PARP1 and further increasing PARP1 protein stabilization.

## Discussion

Multiple deubiquitinating enzymes, including USP1, USP7, and USP10 [23–25], have been reported to play a regulatory role in the development of various cancers. Some deubiquitinating enzymes have also been explored as potential anti-tumor drugs [26, 27]. As a well-known enzyme in this category, USP1 can promote proliferation, invasion, and metastasis of breast cancer and hepatocellular carcinoma through deubiquitination [9, 23]. However, its regulatory role in CCA remains unclear. In this study, we have observed a high expression of USP1 in CCA tissues; indeed, enforced overexpression of USP1 promoted both proliferation and metastasis of CCA cells in vitro and in vivo. Mechanistically, USP1’s positive regulation of PARP1 through deubiquitination at K197 and consequent prevention of its proteasomal degradation was necessary and sufficient for these pro-oncogenic phenotypes. Finally, the levels of both proteins are correlated with each other and with poor survival in CCA patient samples.

Several PARP1 inhibitors have been FDA-approved for the treatment of DNA damage repair (DDR)-deficient cancers including those harboring BRCA1/2, ATM, and RAD51C mutations [28, 29]. These primarily function by enhancing the inherent genomic instability of these cancers, leading to the induction of programmed cell death, as PARP1 is a critical sensor and coordinator of DNA double-strand breaks. However, the role of oncogenic PARP1 functions outside of DDR has been less well studied, and here we provide evidence that PARP1 promotes CCA growth and metastasis in vivo, suggesting that PARP1 could also be a more general target in this cancer type. In future studies, we will assess the pharmacological inhibition of USP1 and/or PARP1 as anti-cancer compounds for CCA.

Our discovery of PARP1 as a primary deubiquitination target of USP1 supported the importance of PTMs in CCA, and thus we also sought additional PTM regulation of the USP1-PARP1 axis. Through multiple functional experiments, we identified GCN5 (also known as KAT2A) as the primary USP1 acetyltransferase, leading to USP1’s positive regulation and enhanced downstream PARP1 deubiquitination. Mechanistically, we pinpointed K130 acetylation as necessary and sufficient for this positive regulation of USP1, through the creation of reciprocal non-acetylable and acetyl-mimetic mutants.

Interestingly, although previous research has identified other USP1 deubiquitination targets as pro-oncogenic, including KPNA2 in breast cancer metastasis [23] and RPS16 in hepatocellular carcinoma [30], these were not identified as USP1 substrates in our CCA model, suggesting that USP1 may serve different functions in different cancer types.

In summary, our findings suggest that USP1 functions as a deubiquitinase to modulate the growth and metastasis of CCA by opposing the ubiquitination-mediated degradation of PARP1. Moreover, we demonstrate that this regulatory mechanism is governed by the acetylation status of USP1 by GCN5. This GCN5-USP1-PARP1 axis provides novel insights into the role of acetyltransferases and deubiquitinases in CCA pathogenesis and may pave the way for developing improved targeted therapies against this deadly disease.

## Materials and methods

### Cell culture and tissue samples

The CCA cell lines RBE, HCCC-9810, HuCC-T1, and human HEK-293T cells were procured from the Cell Bank of the Chinese Academy of Sciences (national repository for authenticated cell cultures located in Shanghai, China). These cells were cultured in DMEM (SH30284.01, HyClone) supplemented with 10% FBS (10099, Gibgo) and 1% penicillin-streptomycin solution (15070063, Thermo Fisher Scientific), maintained at a temperature of 37 °C under an atmosphere containing 5% CO_2_. These cell lines were authenticated by STR before the experiment. Cell lines were regularly tested for mycoplasma contamination using the J66117 Kit (Thermo Fisher Scientific) every 3 months. A total of 65 pairs of CCA and paracancerous tissues were collected from the First Affiliated Hospital of Bengbu Medical College between June 2016 and December 2021, with all patients undergoing surgery without prior radiotherapy or chemotherapy. This study was approved by the Ethics Committee of Bengbu Medical College (no. 2021230).

### Plasmid transfection and lentiviral infection

These plasmids were all constructed with pcDNA3.1(37680, Addgene) by our lab: HA-USP1-1-200, HA-USP1-201-785, HA-USP1-1-400, HA-USP1-401-785, Myc-PARP1-1-779, Myc-PARP1-1-638, Myc-PARP1-1-476, Myc-PARP1-1-203, Myc-PARP1-203-1014, Flag-PARP1, USP1 C90S, His-Ub, His-K6, His-K11, His-K27, His-K29, His-K33, His-K48, His-K63, Myc-PARP1-K197R, Flag-P300, Flag-GCN5, Flag-PCAF, Flag-CBP, Flag-Tip60, HA-USP1-K130Q, HA-USP1-K130R. GST-USP1 and GST-USP1 C90S plasmids were constructed with pGEX-4T-1 (129567, Addgene) by our lab. Following the instructions provided, cells were transfected with Lipofectamine 3000 (L3000075, Thermo Fisher Scientific). Chemical interventions and protein assays were conducted 48 hours post-transfection. The Myc-PARP1, HA-USP1, sh-USP1 and sh-PARP1 plasmid were constructed with Plvx-Puro vector (Bio-114923, Biobw). Then they were introduced into CCA cells via virus transfection. The sequence of sh-USP1 and sh-PARP1 list as Supplementary Table 1. Specifically, the virus was generated in HEK-293T cells using a four-plasmid transfection system (VSVG, pLP1, pLP2, target plasmid) facilitated by Lipofectamine 3000. Then the virus infect CCA or HEK-293T cell line with Polybrene (TR-1003, Sigma-Aldrich). The stable cell line was established through puromycin selection.

### Immunoblotting (IB)

The IB procedure was performed according to the previously described protocol [31]. The antibodies utilized for IB analysis are specified in Supplementary Table 2. In brief, total cellular or tissues protein were extracted using RIPA buffer (R0278, Sigma-Aldrich) supplemented with PMSF protease inhibitor (36978, ThermoFisher Scientific), separated by SDS-PAGE gel electrophoresis and subsequently transferred onto a PVDF membrane (PB9220, ThermoFisher Scientific). The primary antibody was incubated and the secondary antibody was reacted, followed by detection of target bands using a chemiluminescence kit (89880, ThermoFisher Scientific).

### Quantitative real-time polymerase chain reaction (qRT-PCR)

As previously described [31], total RNA was extracted from cells using Trizol (15596026CN, ThermoFisher Scientific) and subjected to PCR amplification with the One Step SYBR® Green RT-qPCR Kit (QR0100, ThermoFisher Scientific) according to the manufacturer’s instructions. The primer sequences used for qRT-PCR are provided in Supplementary Table 3.

### Immunohistochemistry (IHC)

As previously described [31], the CCA slides (*n* = 65) were baked at 60°C for 2 hours, then deparaffifinized and hydrated by dimethylbenzene(1330-20-7, Sigma-Aldrich) and ethanol (64-17-5, Sigma-Aldrich). Antigens were then repaired in citrate buffer (C9999, Sigma-Aldrich). Endogenous peroxidase was blocked with 3% H_2_O_2_ and then slides were blocked with bovine serum albumin (BSA) (A1595, Sigma). Next, the slides were incubated overnight along with the indicated primary antibodies. The next day, the slides were incubated together with the secondary antibodies. Samples were stained by dimethylbenzene (521116, Sigma-Aldrich) and hematoxylin (51275, Sigma-Aldrich), then sealed with neutral gum. All the tissue samples were scanned under a fluorescent microscope. IHC and protein expression scoring were described in previous reports [32].

### Immunofluorescence

CCA cell lines or HEK-293T cell lines cultured on glass coverslips were transfected with the designated plasmids. Subsequently, cells were fixed in 4% paraformaldehyde (FB002, ThermoFisher Scientific) for 20 minutes, permeabilized with 0.2% Triton X-100 (85111, ThermoFisher Scientific) for 5 minutes, and blocked with 3% BSA for 1 hour. Subsequently, cells were incubated with the indicated primary antibodies overnight at 4°C, followed by treatment with fluorescent dye-conjugated secondary antibodies for 1 hour. Cell nuclei were counterstained with DAPI (D9542, Sigma-Aldrich) for 10 min and images were captured using a fluorescence microscope.

### Proximity ligation assay

The PLA assay was conducted according to the previously reported protocol [33]. For analysis of cultured cells, the cells were grown on Lab-Tek II small chamber slides (154534, Nunc) coated with collagen for at least 16 hours. After being washed twice with PBS, they were fixed in a solution of PBS diluted in 3.7% formaldehyde for 15 minutes at room temperature. Subsequently, the slides were washed with TBS (25 mM Tris, 100 mM NaCl, pH 7.4), incubated in a solution of 50 mM NH4Cl and TBS for 10 minutes, washed again with TBS, permeabilized using Triton X-100 (0.1%) for another 15 minutes and finally rinsed once more with TBST (0.05%, Tween-20/TBS). The samples were subsequently sealed with TBST diluted in 0.5% skim milk (232100, BD Biosciences) and incubated at 37 °C for 2 hours in a humidified chamber, followed by overnight incubation at 4 °C with the appropriate antibody. After washing with TBST, neighbor ligation was performed using rabbit PLUS (DUO92002-30RXN, Sigma-Aldrich) and mouse MINUS Duolink in situ PLA kit (DUO92004-30RX, Sigma-Aldrich), following the manufacturer’s instructions. The coverslips were dehydrated, air-dried, and sealed with a Citifluor sealer containing DAPI. In situ PLA reactions and slide blocking were performed as previously described for cultured cells’ immunofluorescence.

### CO-immunoprecipitation (CO-IP)

The supernatant was obtained by lysing the cells according to the IB protocol as previously described. For endogenous IP assay, cell extracts were pre-cleared with Protein A/G beads (80105 G, ThermoFisher Scientific) and control IgG, mixed and incubated at 4 °C for 1 hour. After centrifugation, 0.5 ml of supernatant was added with 3 μg of antibody for protein pull-down. An additional 0.5 ml of supernatant was supplemented with an equivalent amount of homologous IgG antibody as a control and incubated overnight at 4 °C. The following day, 50 μl of Protein A/G Beads (50%) were added to each tube and pulled down for 3 hours at 4 °C. Magnetic beads were washed three times with Tris-buffered saline (TBS, R017R.0000, ThermoFisher Scientific) containing 0.05% Tween-20 (85113, ThermoFisher Scientific) for 5 minutes each. Bound proteins were washed with 2× Laemmli buffer (S3401-10VL, Sigma-Aldrich) by centrifugation at room temperature for 10 minutes. For IP of overexpressed tagged fusion proteins, cell extracts were pre-cleared with Protein A/G beads and then incubated overnight at 4 °C with either anti-MYC agarose beads (20168, ThermoFisher Scientific) or anti-HA magnetic beads (88836. ThermoFisher Scientific). IB analysis was performed using antibodies against HA or Myc.

### GST Pull-down assay

The GST pull-down assay was employed to detect protein-protein interactions. Briefly, the Myc-PARP1 plasmid was transfected into HEK-293T cells, followed by cell lysis with GST lysate and subsequent collection of proteins. The USP1 and USP1 C90S sequences were cloned into the pGEX-4T-1 vector to obtain GST-USP1 and GST-USP1 C90S plasmids, respectively. These plasmids were then transferred into E. Coli BL21 (DE3, EC0114, ThermoFisher Scientific) for protein expression induced by IPTG (100 μM, 34060, ThermoFisher Scientific). After collection and sonication of the E. coli cells, purified GST-USP1 or GST-USP1 C90S proteins were obtained using complete GST-Tag purification resin (08778850001, Roche). The protein was expressed and purified following the manufacturer’s instructions, utilizing BeyoGold™ GST-tag purification resin (P2251, Beyotime) for purification. The beads were washed with GST pull-down binding buffer (50 mM Tris-HCl, 200 mM NaCl, 1 mM EDTA, 1% NP-40, 1 mM DTT, 10 mM MgCl2; pH 8.0), then incubated with purified GST-USP1 or GST-USP1 C90S in complex with Myc-PARP1 on a rotary windmill at 4 °C for 4 hours. Finally, the beads were washed and subjected to western blot analysis.

### In vivo and in vitro deubiquitination assay

Cells were treated with 20 μM MG132 (M7449, Sigma-Aldrich) for 8 hours, followed by lysis using RIPA buffer containing 1% SDS and mild sonication. The resulting cell lysates were diluted to a final concentration of 0.2% SDS using an SDS-free lysis solution prior to IP with a PARP1 primary antibody at 4 °C. Ubiquitination levels were subsequently analyzed via Western blot. In vitro deubiquitination assays were performed as follows: HEK-293T cells were co-transfected with Myc-PARP1 and His-Ub expression vectors for 24 hours. The cells were then treated with MG132 (20 μM) for 8 hours, followed by IP of Myc-PARP1 using Myc affinity magnetic beads. The resulting immunoprecipitates were washed three times with a wash buffer before competitive elution of Myc-PARP1 was achieved using Pierce™ c-Myc peptide (F4799, Sigma-Aldrich) in Pierce™ IP lysis Buffer (87788, ThermoFisher Scientific). Similarly, the HA synthetic peptide (26184, ThermoFisher Scientific) competitively elutes HA-USP1 or HA-USP1 C90S bound to HA magnetic beads. The purified HA-USP1 or HA-USP1 C90S protein (200 ng) was respectively incubated with recombinant PARP1 protein (200 ng) in deubiquitination buffer (50 mmol/L Tris-HCl pH 8.0, 50 mmol/L NaCl, 1 mmol/L EDTA, 10 mmol/L DTT and 5% glycerol) at a temperature of 37 °C for 2 hours. The ubiquitination levels of Myc-PARP1 were analyzed by Western blot.

For in vitro DUB Activity Assay: Purified HA-USP1 (0.05 μg/μL) was combined with K48-linked Di-ubiquitin (U2701, KS-V Peptide, Hefei, China) at a concentration of 0.1 μg/μL in the presence of the compound and incubated in a DUB reaction buffer (50 mM HEPES, pH 7.5, 100 mM NaCl, 2 mM TCEP) at 37 °C for 1.5 hours. The reaction was terminated by adding 2× Tricine sample buffer (Bio-Rad, Hercules, CA, USA) and incubating at 40 °C for 20 minutes. Subsequently, the samples were loaded onto a Tris-Tricine gel (Bio-Rad) for IB analysis using a specific antibody targeting ubiquitin.

Other reagent used for cell line: (MB-3, M2449), Cycloheximide (CHX, C7698), Chloroquine (CQ, C6629) were purchased from Sigma-Aldrich.

### Mass spectrometry

The Ubiquitin Modified Quantitative Protein Genomics for CCA cell line, as previously described [34], the timsTOF Pro (Bruker Daltonics) mass spectrometry performed by PTM Bio (PTM Bio Inc., Hangzhou, China). Briefly, proteins from the cell line were extracted and hydrolyzed with trypsin at a 1:50 trypsin-to-protein mass ratio. Modified peptides were then enriched using Pan-antibody-based PTM. The peptides were analyzed by capillary electrospray ionization and subsequently subjected to timsTOF Pro mass spectrometry. The resulting MS/MS data were processed using the MaxQuant search engine (v.1.6.15.0). For deubiquitin site mass spectrometry of cell lines, USP1 was co-transfected into RBE cells. After collecting the cell lysates 48 hours later, Gel-LS-MS/MS sequencing and data analysis were performed to identify the ubiquitination sites of PARP1 using MASCOT engine (Matrix Science, London, UK; version 2.2) embedded in Proteome Discoverer 1.4 (Thermo Electron, San Jose, CA.).

### Transwell assay

The matrix gel (356234, BD Biosciences) was thawed overnight at 4 °C and transferred to an ice box prior to the experiment. The gun tip, centrifuge tube, and 24-well plate with transwell (353097, Falcon) were pre-cooled in the ice box while diluting the matrix gel with pre-cooled serum-free medium. The substrate gel was then added vertically into the upper chamber of the Transwell and spread evenly on its bottom surface. Incubate in the incubator for 2 hours to polymerize the matrix gel into a thin film. After adding 100 µL of serum-free medium to each well, incubate for another 30 minutes. Then, add 200 µL of cell suspension (with a density of 2.5 × 10^5^/mL) to the upper chamber of the transwell and place it on top of the lower chamber containing 500 µL medium with 10% FBS. Incubate for 24 hours, then remove the stromal gel and cells from the upper chamber with a cotton swab. Fix the cells at the bottom of the chambers with 4% paraformaldehyde for 30 minutes, and stain with 0.1% crystalline violet (548-62-9, Sigma-Aldrich) for 5 minutes. The cells were subjected to microscopic examination and quantification.

### Cell clone assay

In brief, 500 cells were seeded per well in six-well plates and cultured for 2 weeks. The cells were then fixed with 4% paraformaldehyde for 20 minutes, washed with PBS, and stained with Giemsa (51811-82-6, Sigma-Aldrich) for 15 minutes. The clones were photographed with camera and the number of clones was calculated.

### Apoptosis assay

Annexin V-FITC/PI apoptosis assay kit (FAK012.20, NEOBIOSCIENCE, shanghai, China) was used to detect cell apoptosis, following the manufacturer’s protocol. Cells in different groups were resuspended at a density of 5 × 10^5^ cells/mL, then incubate with Annexin V-FITC and propidineiodide (PI), and a flow cytometry analysis (Guava easyCyte HT, Millipore, Billerica, MA, USA) was then performed.

### Animal experiment

For the tumor formation assay, 2.5 × 10^6^ RBE or HuCC-T1 cells transfection with indicated plasmid, were suspended in 200 μL of PBS and subcutaneously implanted into 6-week-old BALB/c mice (Shanghai Model Organisms Center, Inc, China. Each group was randomly assigned to six mice) to monitor tumor development. After tumor formation, the tumor volume was measured every three days. Tumor volume was calculated by the formula: Volume = (Length × width^2^)/2. After 4 weeks, the mice were euthanized to obtain tumors for observation of tumor weight. For mice tail intravenous injection assay: 2.5 × 10^6^ cells (sh-USP1, sh-USP1, and overexpressed PARP1, overexpressed USP1, overexpressed USP1, and sh-PARP1 cell line) were dissolved in 200ul PBS and injected into the tail vein of 6-week-old BALB/c mice, and lung metastases were observed once every 2 weeks under magnetic resonance. Mice were euthanized when they met the institutional euthanasia criteria for tumor size or overall health condition. The remaining tissues were fixed in 10% buffered formalin (15512-1L-R, Sigma-Aldrich) overnight, washed with PBS, and transferred to 70% ethanol. Embedded in paraffin, sectioned, and stained with hematoxylin and eosin (H&E, C0105S, Beyotime). All tumor tissues were obtained and confirmed as CCA or CCA pulmonary metastasis by HE staining. The study was approved by the Animal Ethics Committee of Bengbu Medical College (no. 2021298).

All animals utilized in our experiment were housed within specific-pathogen-free (SPF) animal facilities, where they had access to food and water ad libitum and were maintained under a 12-hour light:dark cycle with controlled temperature conditions. Animal studies were conducted in a gender- and age-matched manner using littermates for each experimental group. The animal assays adhered strictly to governmental and international guidelines governing the ethical treatment of animals.

### Statistical analysis

The cell line experiments were repeated at least three times. The data obtained from the appeal trials were subjected to statistical analysis using SPSS 21.0 software and GraphPad Prism version 8.0. Each set of data was presented as mean ± SD. If the data followed a normal distribution and Chi-squared, unpaired *t* test was employed for comparing means between two groups, while one-way ANOVA was used for analyzing data with three or more groups. The Kaplan-Meier method was employed to assess patients’ survival rates, while the chi-square test and linear regression analysis were utilized to statistically analyze the correlation between USP1 and PARP1. The *P* value <0.05 indicates significant differences.

### Supplementary information

Supplementary table 1-3

supplementary table 4: mass spectrometry

supplementary table 5: mass spectrometry

supplementary figure legend

Supplementary Figure 1

Supplementary Figure 2

Supplementary Figure 3

Supplementary Figure 4

Original blot

Data Set

checklist

## Data Availability

All data relevant to the study are included in the paper or uploaded as supplementary material. The data and sources associated with this study are available from the corresponding author upon reasonable request.
