# Supplementary material for: USP1 promotes cholangiocarcinoma progression by deubiquitinating PARP1 to prevent its proteasomal degradation
Source: Cell Death Dis. 2023 Oct 11;14(10):669. doi: 10.1038/s41419-023-06172-6 (PMC10567853; doi:10.1038/s41419-023-06172-6)
Supplement: Supplementary file 1 — Supplementary table 1-3 [file 41419_2023_6172_MOESM1_ESM.docx]

Supplement table 1: Plasmid information.

| **Plasmids name** | **Target sequence (5'to3')** |
| --- | --- |
| USP1 Sh#1 | GCATTACACTGCTTCTGTT |
| USP1 Sh#2 | CTTGGAATGTGAAAGTTTA |
| USP1 Sh#3 | ACAAAAGAAATCAAGAGTT |
| Negative Control | TACAAACGCTCTCATCGACAAG |
| PARP1 Sh#1 | CGGTGATCGGTAGCAACAA |
| PARP1 Sh#2 | TGGACCAAGTGTATGGTCA |
| PARP1 Sh#3 | AGGAGGAAGGTATCAACAA |

| **Supplementary Table 2:** The antibodies information. | | | | | | | | | | | |
| --- | --- | --- | --- | --- | --- | --- | --- | --- | --- | --- | --- |
| **Antigen** | **Species** | **Source** | **Catalog#** | **Dilution** | **Application** | **Dilution** | **Application** | **Dilution** | **Application** | **Dilution** | **Application** |
| USP1 | Rabbit | Proteintech | 14346-1-AP | 1:800 | WB | 1:80 | IP | 1：200 | IHC | 1:200 | IF |
| PARP1 | Rabbit | Proteintech | 13371-1-AP | 1:1200 | WB | 1:100 | IP | 1：200 | IHC | 1:200 | IF |
| Myc-tag | Rabbit | Cell Signaling Technology | 2278 | 1:1000 | WB | 1:50 | IP | - | - | 1:50 | IF |
| HA-tag | Rabbit | Cell Signaling Technology | 3724 | 1:1000 | WB | 1:50 | IP | - | - | 1:50 | IF |
| His-tag | Rabbit | Cell Signaling Technology | 12698 | 1:1000 | WB | 1:50 | IP | - | - | - | - |
| β-actin | Rabbit | Cell Signaling Technology | 4970 | 1:5000 | WB | - | - | - | - | - | - |
| IgG-tag | Rabbit | Cell Signaling Technology | 14708 | 1:1000 | WB | 1:50 | IP | - | - | 1:200 | IF |
| GST-tag | Mouse | Proteintech | 66001-2-Ig | 1:5000 | WB |  |  | - | - | - | - |
| Flag-tag | Mouse | Sigma-Aldrich | F1804 | 1:1000 | WB | 1:50 | IP | - | - | - | - |
| Ubiquitin (E6K4Y) XP® Rabbit mAb | Rabbit | Cell Signaling Technology | 20326 | 1:1000 | WB | - | - | - | - | - | - |
| Acetylated-Lysine Antibody | Rabbit | Cell Signaling Technology | 9441 | 1:1000 | WB | - | - | - | - | - | - |
| GCN5 | Mouse | Sigma-Aldrich | MABE1791 | 1:1000 | WB | 1:50 | IP | - | - | 1:50 | IP |
| GNAI1 | Rabbit | Sigma-Aldrich | SAB2100936 | 1:1000 | WB | - | - | - | - | - | - |
| CBX5 | Rabbit | Sigma-Aldrich | SAB5701041 | 1:1000 | WB | - | - | - | - | - | - |
| RFC4 | Rabbit | Proteintech | 10806-1-AP | 1:5000 | WB | - | - | - | - | - | - |
| SCAF1 | Rabbit | GeneTex | GTX55206 | 1:800 | WB | - | - | - | - | - | - |
| PDAP1 | Mouse | Sigma-Aldrich | SAB1403111 | 1:800 | WB | - | - | - | - | - | - |
| MEAF6 | Rabbit | Thermo Fisher Scientific | PA5-116390 | 1:1500 | WB | - | - | - | - | - | - |
| FAR1 | Rabbit | Proteintech | Ag9050 | 1:1000 | WB | - | - | - | - | - | - |
| PCNP | Rabbit | Thermo Fisher Scientific | PA5-70315 | 1:1000 | WB | - | - | - | - | - | - |
| HMGN2 | Rabbit | Proteintech | 10953-1-AP | 1:1000 | WB | - | - | - | - | - | - |
| HRP-labeled Goat Anti-Mouse IgG(H+L) | Goat | Thermo Fisher Scientific | 31430 | 1:1000 | WB | - | - | - | - | - | - |
| HRP-labeled Goat Anti-Rabbit IgG(H+L) | Goat | Thermo Fisher Scientific | 31460 | 1:1000 | WB | - | - | - | - | - | - |
| Alexa Fluor 350-labeled Goat Anti-Rabbit IgG (H+L) | Goat | Thermo Fisher Scientific | A-11046 | 1:1000 | WB | - | - | - | - | 1:300 | IF |
| Alexa Fluor 488-labeled Goat Anti-Mouse IgG(H+L) | Goat | Thermo Fisher Scientific | A-11001 | 1:1000 | WB | - | - | - | - | 1:300 | IF |

Table 3. The primer sequences used for qRT-PCR

| USP1 | Forward  5’-TTCGGTTGAACAGCTCCAGG-3’ |
| --- | --- |
|  | Reverse  5’-GGGTTGAGTTCCCTCAGTGT-3’ |
| PARP1 | Forward 5’-GGCGATCTTGGACCGAGTAG-3’ |
|  | Reverse  5’-GCGATCTTGGACCGAGTAG-3’ |
| β-actin | Forward 5’--GATGAGATTGGC ATGGCTTT-3’  Reverse  5’--CACCTTCACCGT TCCAGTTT-3’ |
