# Supplementary material for: USP1 promotes cholangiocarcinoma progression by deubiquitinating PARP1 to prevent its proteasomal degradation
Source: Cell Death Dis. 2023 Oct 11;14(10):669. doi: 10.1038/s41419-023-06172-6 (PMC10567853; doi:10.1038/s41419-023-06172-6)
Supplement: Supplementary file 4 — supplementary figure legend [file 41419_2023_6172_MOESM4_ESM.docx]

**Supplementary Figure 1. Identification of potential substrates of USP1 by tissue proteomics.**

A: Mass-spectrometry analysis of an PARP1 peptide in USP1 precipitate. Using an anti-USP1 antibody to pull down USP1 and its interacting proteins in the over-expression USP1 RBE cell lysates.

B: HEK-293T cells were transfected with plasmids encoding HA-tagged USP1 and Myc-tagged PARP1. After fixation, in situ PLA for HA/Myc was performed with anti-HA and anti-Myc antibodies. The PLA-detected proximity (PROX) complexes are represented by the fluorescent rolling circle products (red dots). Scale bars, 10 μm.

C: HEK-293T cells were co-transfected with HA-USP1 and Myc-tagged FL PARP1 or its deletion mutants, and cell lysates were analyzed by IP with Myc beads followed by IB analysis with antibodies against HA and Myc.

**Supplementary Figure 2. Knockdown efficiency of USP1 shRNAs.**

A-B: After USP1 knockdown in HuCC-T1 (A) and HCCC-9810 (B) cell lines, the efficiency of knockdown by three independent shRNAs was assessed through IB analysis.

C: The mRNA levels of USP1 and PARP1 were quantified by qRT-PCR following the overexpression of USP1 in the RBE cells.

The data were obtained from three independent biological replicates and are presented as mean ± SD. An unpaired two-tailed t-test was performed(C). *** p<0.001，# p>0.05.

**Supplementary Figure 3. USP1 promote CCA proliferation, invasion, and metastasis through PARP1.**

A: After silencing PARP1 in HuCC-T1 cells, the efficacy of knockdown was evaluated using IB analysis with three independent shRNAs. Following overexpression of PARP1 in RBE cells, the efficiency of overexpression was assessed by IB analysis.

B: Colony assay was assessed in HCCC-9810 transfected with shCtrl, USP1-sh1 or USP1-sh2, reconstituted with PARP1. Representative images are presented in left, while the relative number of clones is quantified in right.

C: The Transwell assay was performed on HuCC-T1 cells transfected with shCtrl, USP1-sh1 or USP1-sh2, and reconstituted with PARP1. Representative images are presented in left, while the relative number of migrated cells was quantified in right.

D: The apoptosis of RBE and HuCC-T1 in indicated group were detected by flow Cytometric Analysis.

Data are from three independent biological repeats and presented as mean ± SD. ***P < 0.001, **p<0.005. One-way ANOVA with Dunnett’s post test.

**Supplementary Figure 4. Potential acetylation sites of USP1 in database and co-localization of USP1 and GCN5.**

A: Analysis of proteomic databases (PhosphoSitePlus) indicates that K130 of USP1 is potentially acetylated.

B: HEK-293T cells were fixed and stained with HA antibody (Green) and Flag antibody (Red). Nuclei were stained with DAPI (blue). Scale bar: 10 μm.

C:IB analysis the expression of GCN5 in 14 CCA samples.

D:Representative images of IHC staining of GCN5 on tissue of CCA and paracancerous (n = 14). Scale bars are indicated.
