# Supplementary figures and images for: USP1 promotes cholangiocarcinoma progression by deubiquitinating PARP1 to prevent its proteasomal degradation
Source: Cell Death Dis. 2023 Oct 11;14(10):669. doi: 10.1038/s41419-023-06172-6 (PMC10567853; doi:10.1038/s41419-023-06172-6)

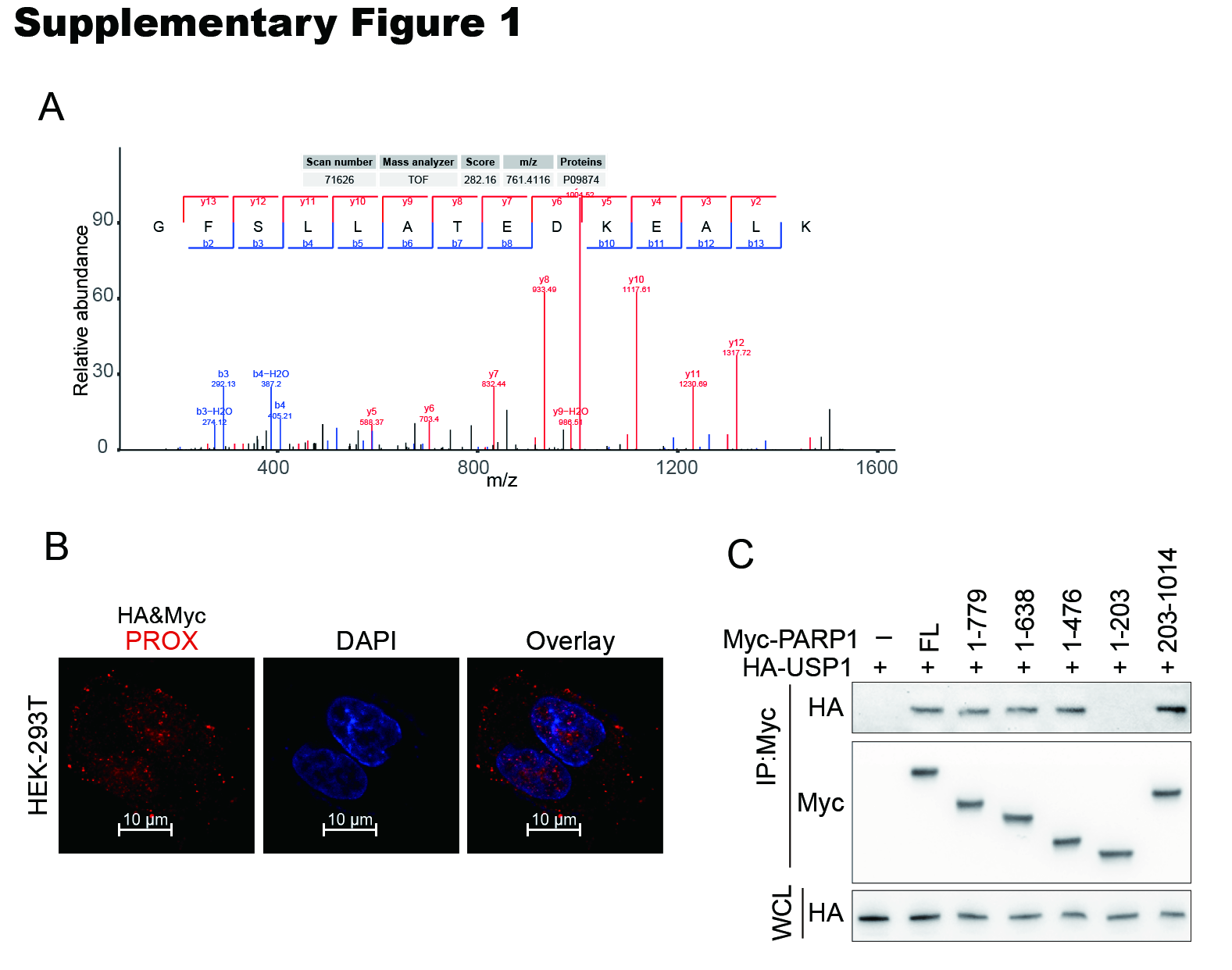

Supplement: Supplementary file 5 — Supplementary Figure 1 [file 41419_2023_6172_MOESM5_ESM.tif]

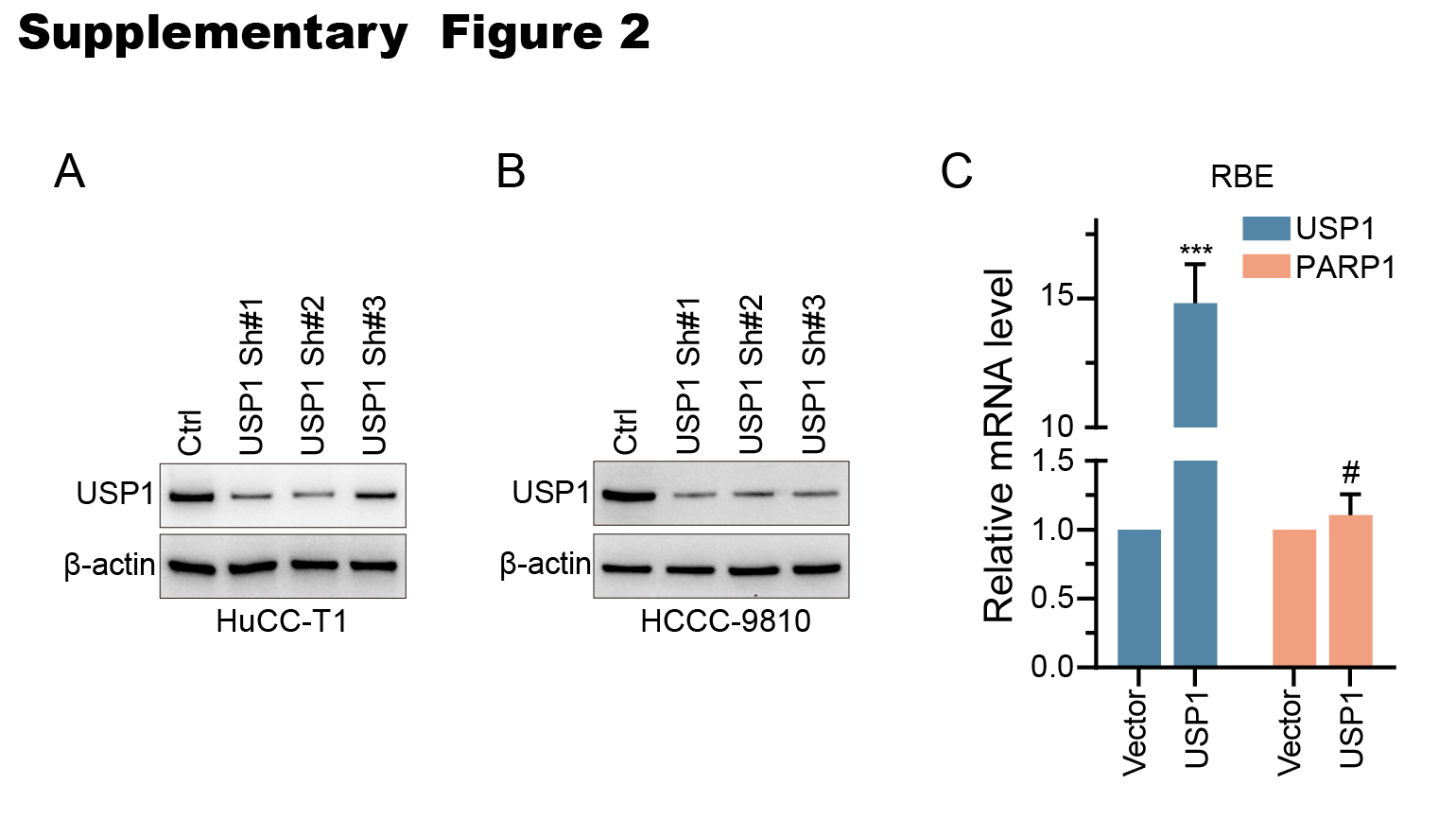

Supplement: Supplementary file 6 — Supplementary Figure 2 [file 41419_2023_6172_MOESM6_ESM.tif]

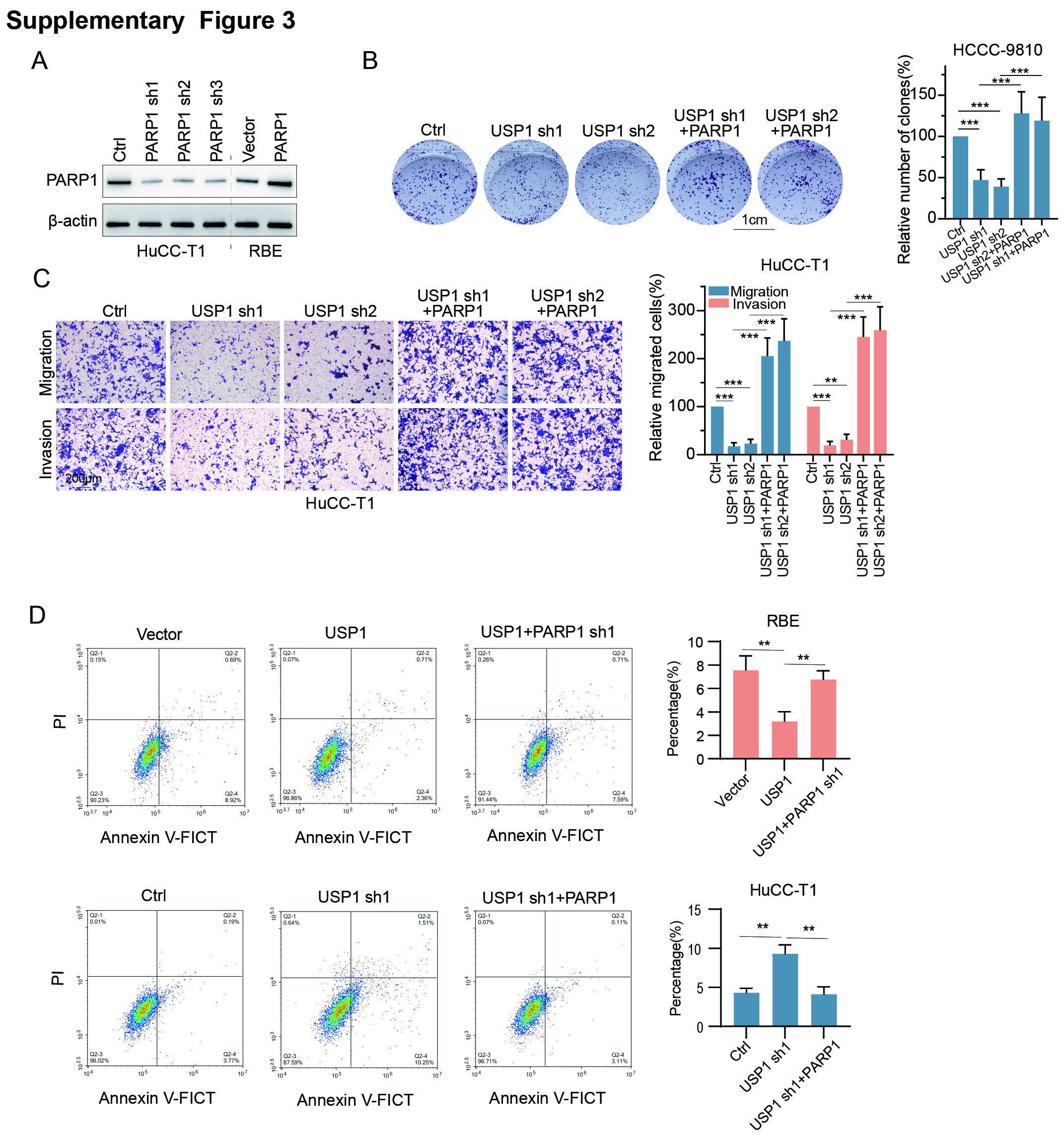

Supplement: Supplementary file 7 — Supplementary Figure 3 [file 41419_2023_6172_MOESM7_ESM.tif]

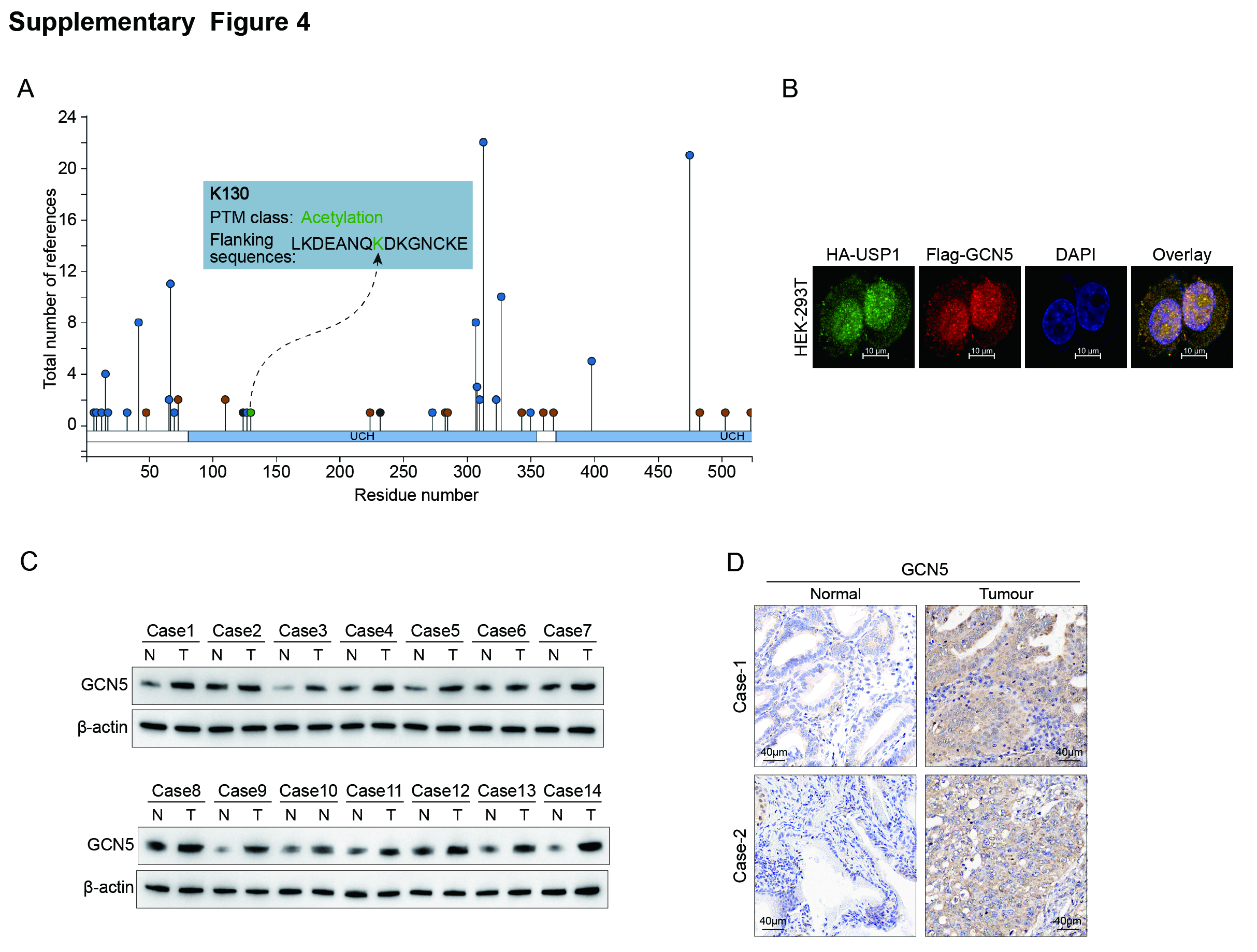

Supplement: Supplementary file 8 — Supplementary Figure 4 [file 41419_2023_6172_MOESM8_ESM.tif]

Figure 1

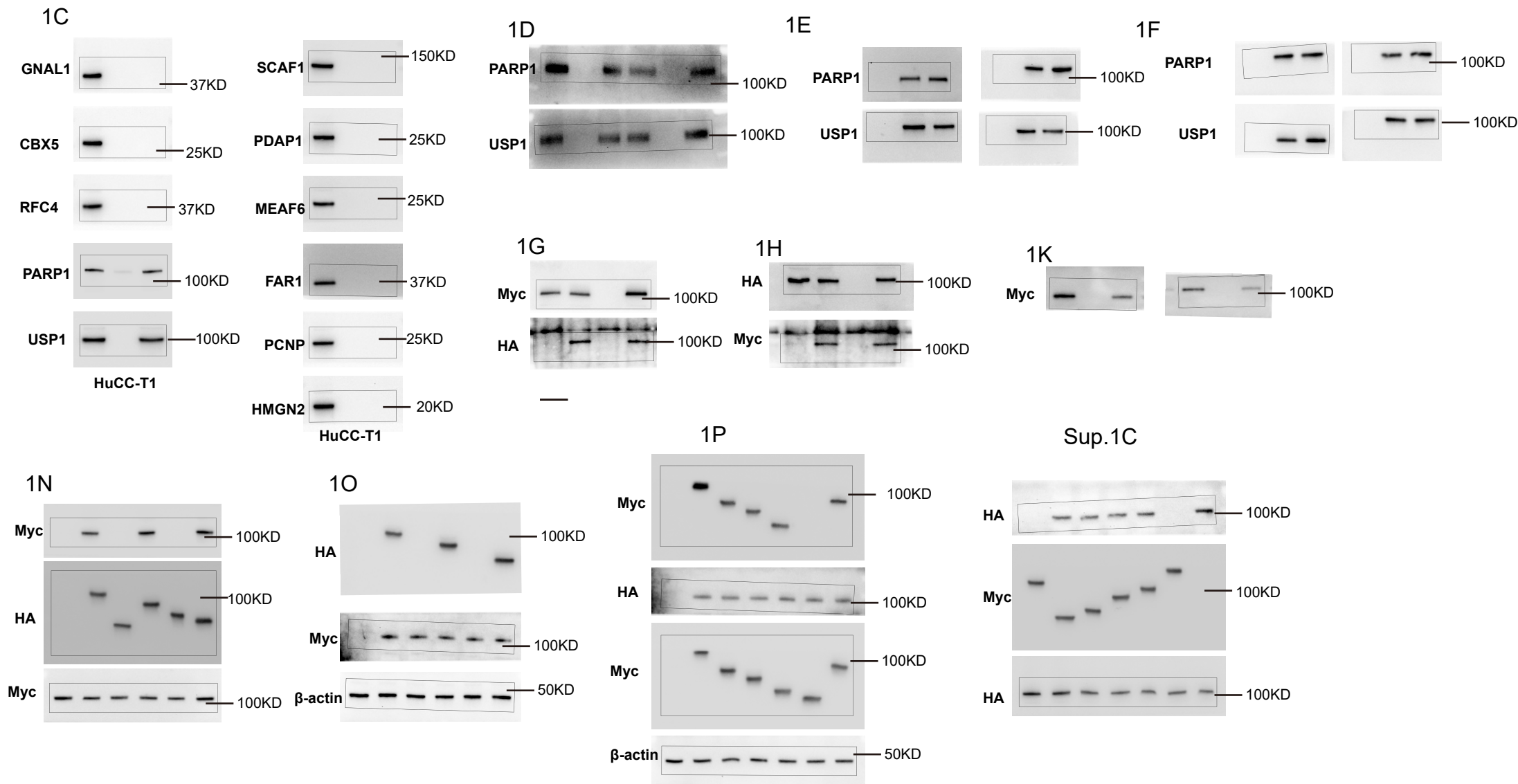

Figure 2

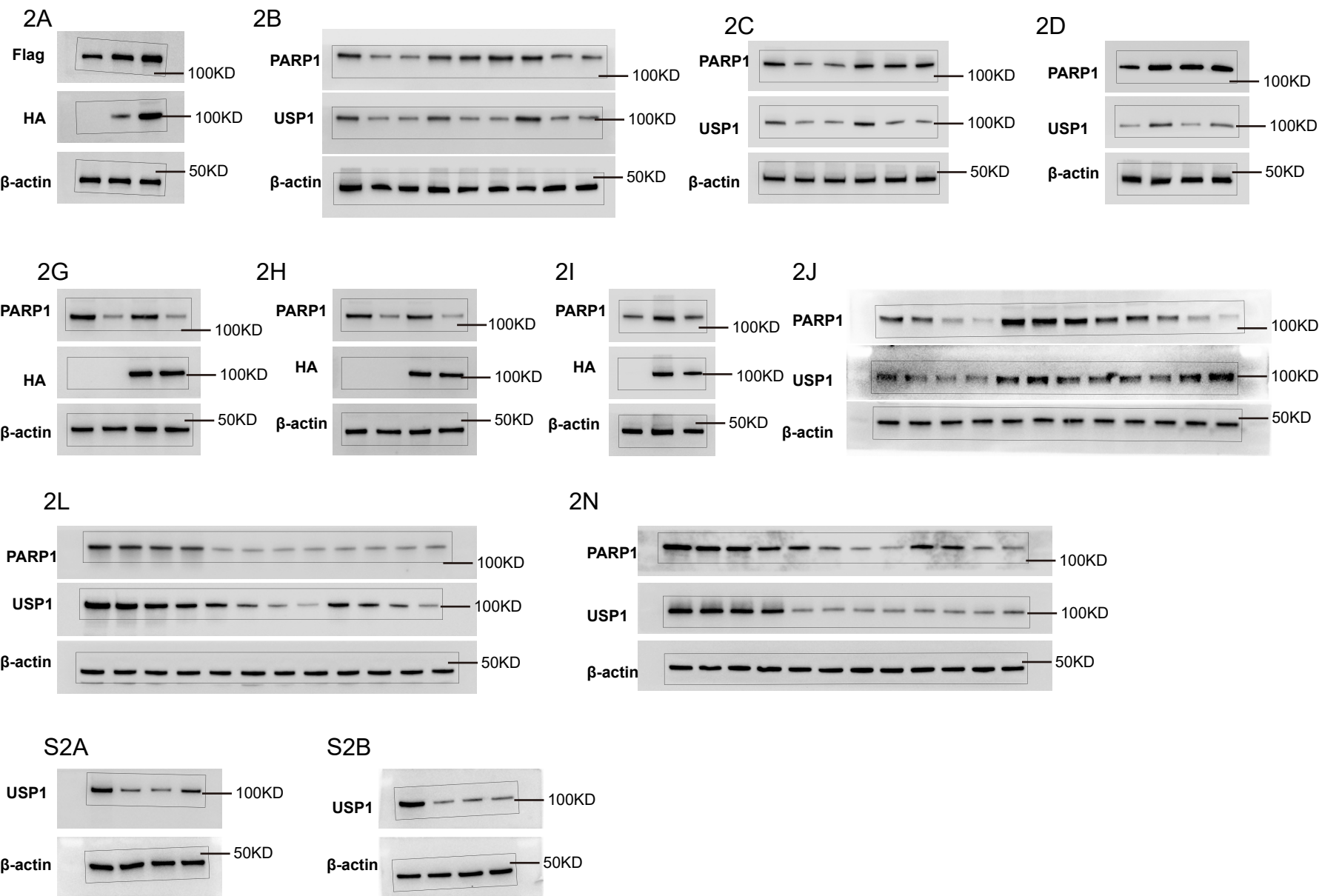

Figure 3

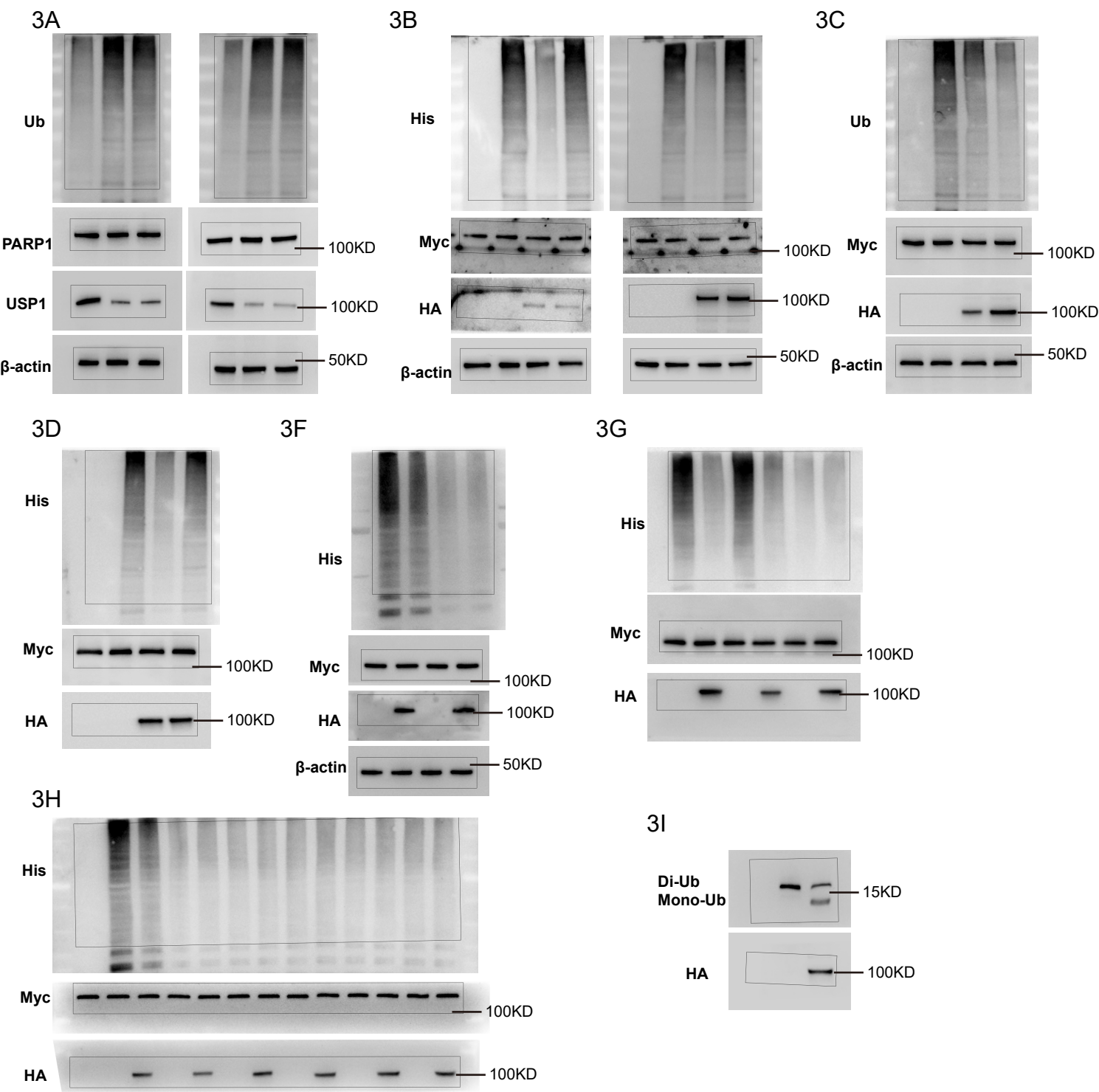

Figure 4

Figure S 3A

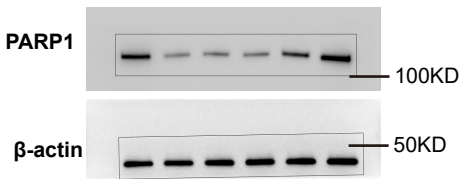

Figure S 4C

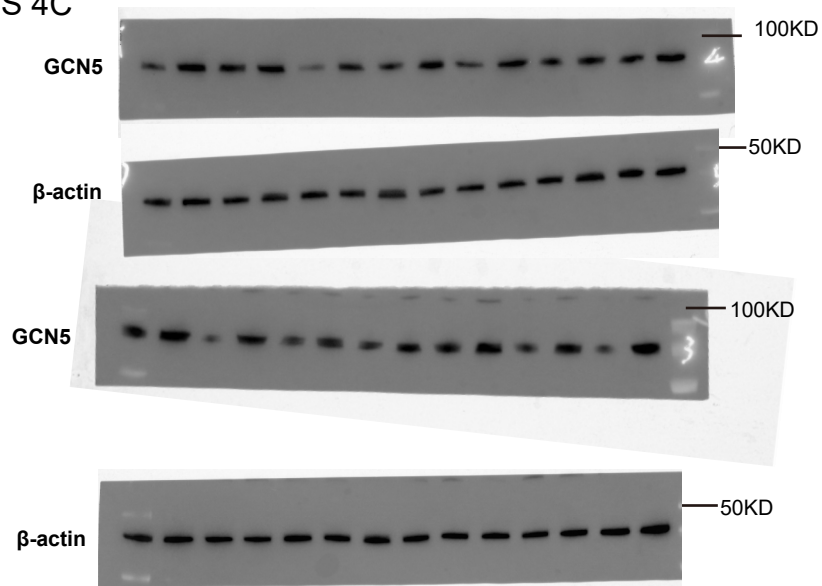

Figure 5

5B

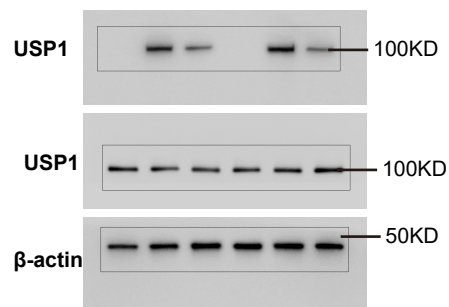

5C

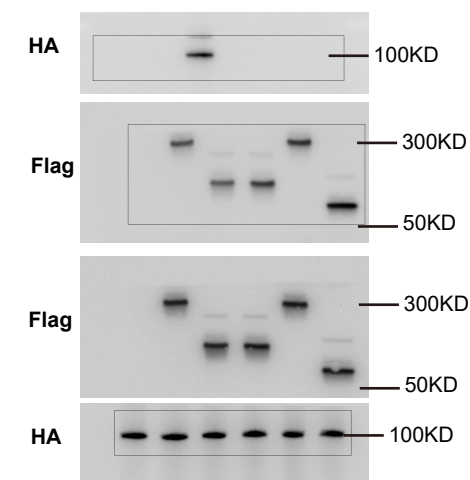

5D

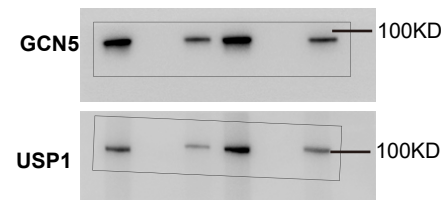

5F

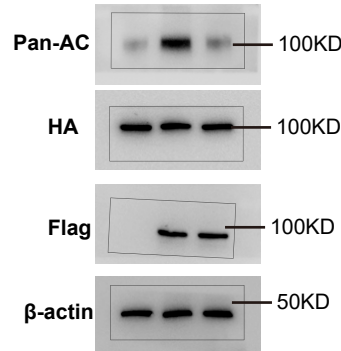

5J

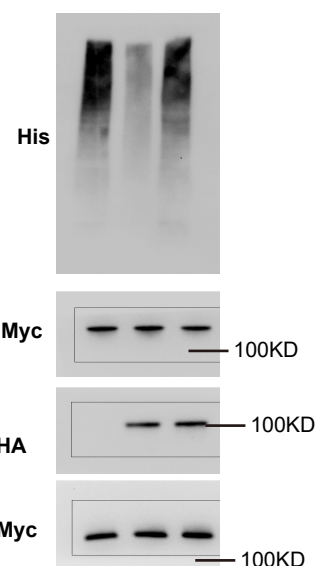

5G

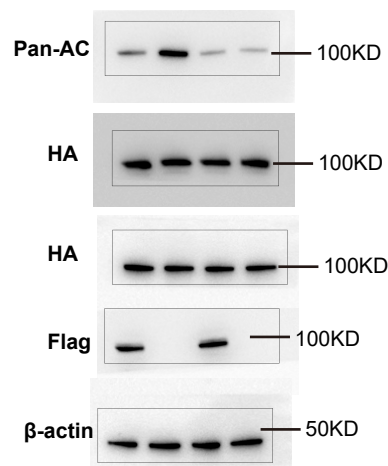

5H

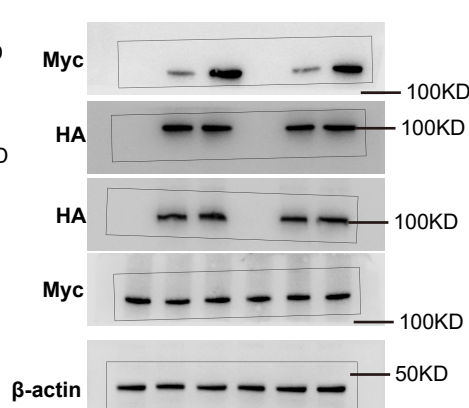

5I

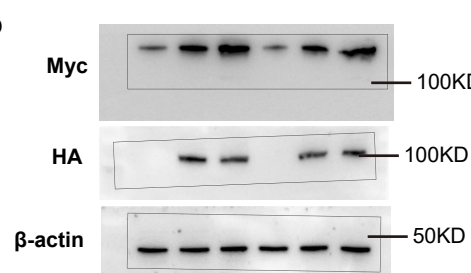

5K

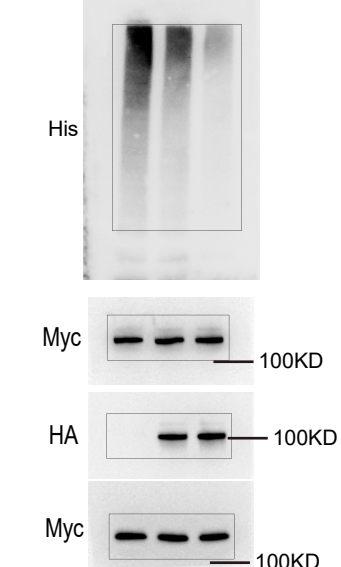

5L

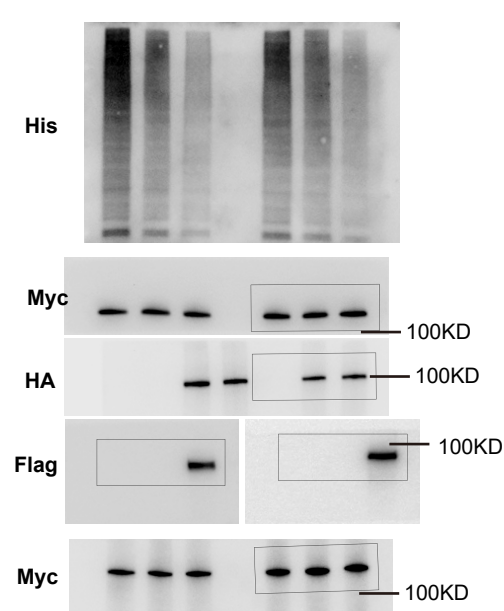

5M

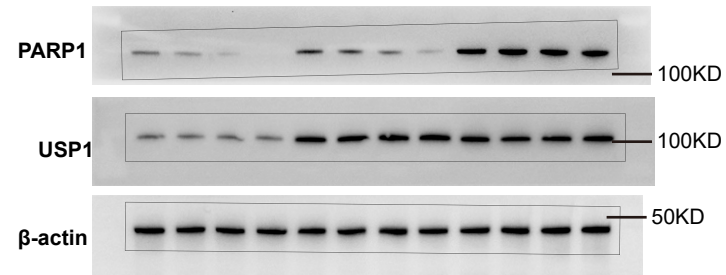

5O

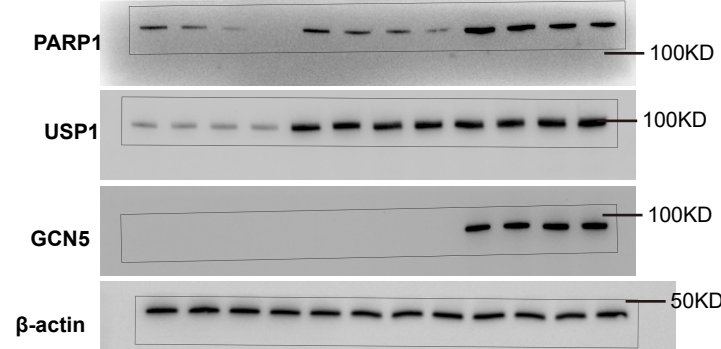

Figure 6

6A

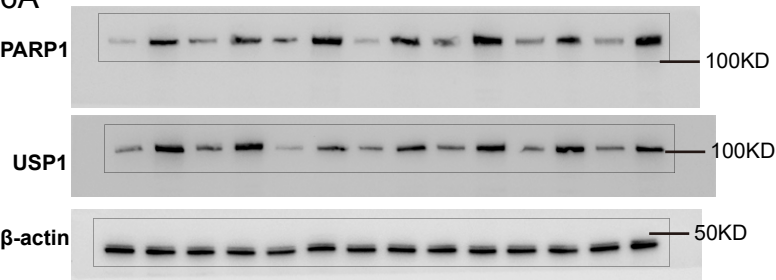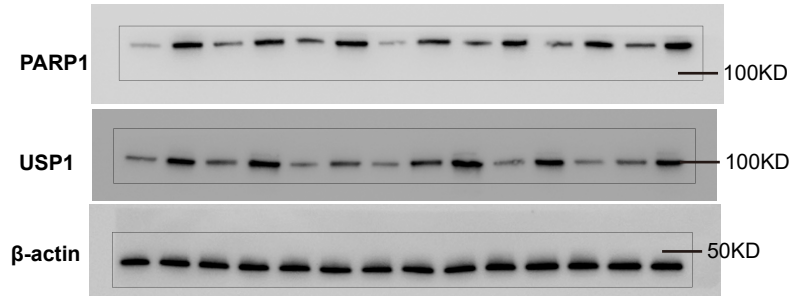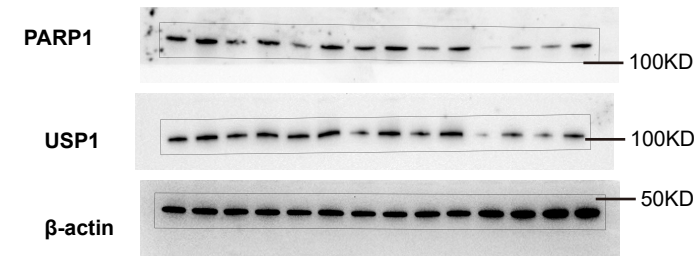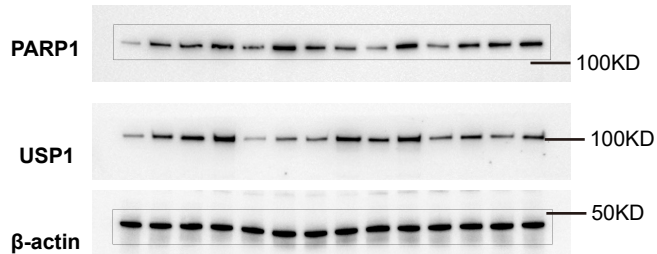

Supplement: Supplementary file 9 — Original blot [file 41419_2023_6172_MOESM9_ESM.pdf]
